# Low-dose of yeast beta-glucan on respiratory symptoms and psychological well-being in moderately stressed adults

**DOI:** 10.1016/j.isci.2026.115120

**Published:** 2026-02-25

**Authors:** Nur Nadia Mohamad Habibullah, Munirah Ismail, Norhayati Ibrahim, Shirley Gee Hoon Tang, Hanif Farhan Mohd Rasdi, Mohamed Faisal Abdul Hamid, Kalavathy Ramasamy, So Fie Tan, Suzana Shahar

**Affiliations:** 1 Dietetics Programme, Centre for Healthy Ageing and Wellness, Faculty of Health Sciences, Universiti Kebangsaan Malaysia, Kuala Lumpur 50300, Malaysia; 2 Clinical Psychology Programme, Centre for Healthy Ageing and Wellness (H-CARE), Faculty of Health Sciences, Universiti Kebangsaan Malaysia, Kuala Lumpur 50300, Malaysia; 3 Biomedical Science Programme, Centre for Toxicology and Health Risk Studies (CORE), Faculty of Health Sciences, Universiti Kebangsaan Malaysia, Kuala Lumpur 50300, Malaysia; 4 Occupational Therapy Programme, Centre for Rehabilitation and Special Needs Studies (ICaRehab), Faculty of Health Sciences, Universiti Kebangsaan Malaysia, Kuala Lumpur 50300, Malaysia; 5 Respiratory Unit, Faculty of Medicine, Universiti Kebangsaan Malaysia (UKM), Cheras, Kuala Lumpur 56000, Malaysia; 6 Collaborative Drug Discovery Research (CDDR) Group, Faculty of Pharmacy, Universiti Teknologi MARA (UiTM) Cawangan Selangor, Kampus Puncak Alam, Bandar Puncak Alam, Puncak Alam, Selangor Darul Ehsan 42300, Malaysia; 7 RB (Health) Malaysia Sdn Bhd, Menara 1 Sentrum, Jalan Sambathan, Kuala Lumpur 50470, Malaysia

**Keywords:** Health sciences, Medicine, Psychiatry, Psychology

## Abstract

Yeast beta-glucan (YBG) is an immunomodulatory polysaccharide that enhances host defense, but optimal dosing for respiratory and psychological benefits requires investigation. This 12-week randomized, double-blind, placebo-controlled trial (ISRCTN 48336189) enrolled 198 moderately stressed adults (PSS-10 scores: 14–26) assigned to placebo, low-dose YBG (120 mg), or moderate-dose YBG (204 mg) daily. The primary outcome was upper respiratory tract infection symptom severity measured by WURSS-21. Both YBG doses improved symptoms compared with placebo (adjusted mean change, 95% CI: placebo +3.42 [0.91 to 5.93]; 120 mg −2.27 [−4.82 to 0.28]; 204 mg −3.51 [−6.06 to −0.96]), with only 204 mg exceeding the minimal clinically important difference. Both doses reduced negative mood states (*p* < 0.001), with no safety concerns observed. These findings suggest that YBG may support respiratory and emotional health in stressed populations, with 204 mg demonstrating the most consistent clinically meaningful effects.

## Highlights

• Yeast beta-glucan reduced URTI symptom severity
• Moderate-dose yeast beta-glucan improved mood and vitality
• Yeast beta-glucan was safe with stable metabolic biomarkers

## Graphical abstract

**Figure:**
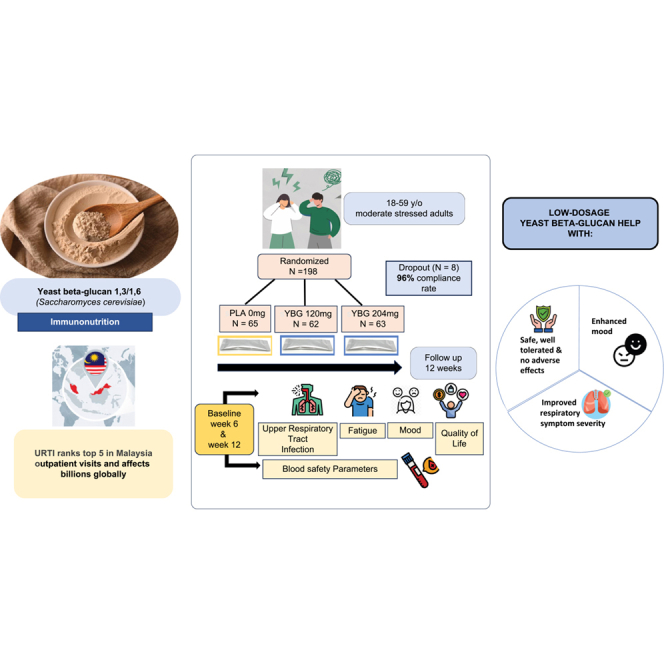

## Introduction

Upper respiratory tract infections (URTIs) are among the most prevalent infectious diseases worldwide, imposing a substantial economic and public health burden through increased absenteeism, reduced productivity, and elevated healthcare expenditure.^1^^,^^2^^,^^3^ In Southeast Asia, URTIs constitute a significant proportion of outpatient consultations and antibiotic prescriptions, particularly among working-age adults.^4^ Consistent with global trends, Malaysia continues to report respiratory infections as one of the top five causes of outpatient morbidity, with URTIs accounting for over one-quarter of annual primary care visits.^5^ The region’s tropical climate, characterized by alternating dry and rainy seasons, together with the influence of climate change, further amplifies infection risk by promoting viral persistence and transmission.^6^^,^^7^ These environmental factors, combined with chronic psychological or physiological stress, can suppress immune function, thereby increasing susceptibility to recurrent infections.^8^

The concept of immunonutrition, in which refers to the strategic use of specific nutrients to modulate immune function has gained growing attention as a non-pharmacological approach to enhance host defense and immune resilience.^9^ Among the most extensively studied immunonutrients is yeast beta-glucan (YBG), a beta-1,3/1,6-linked polysaccharide derived from *Saccharomyces cerevisiae*.^10^^,^^11^ YBG interacts with pattern recognition receptors such as dectin-1 and complement receptor 3 (CR3) on immune cells, stimulating both innate and adaptive responses, enhancing pathogen recognition, and promoting efficient immune regulation.^12^ Preclinical and clinical evidence have demonstrated that YBG can reduce infection incidence and severity, attenuate inflammatory responses, and strengthen immune competence in healthy and stressed populations.^13^^,^^14^^,^^15^

Meta-analyses and controlled trials^14^^,^^16^ further demonstrate that YBG supplementation effectively decreases the incidence, duration, and severity of URTIs in healthy adults while also improving mood and reducing fatigue, suggesting broader psychoneuroimmune benefits.^17^ Collectively, these findings indicate that YBG may serve as a supportive dietary component for maintaining immune function and mitigating stress-related susceptibility to infection. However, most clinical trials to date have focused on moderate to high doses of YBG (≥250 mg/day) over short to moderate durations (ranging from 7 days to 3 months).^18^^,^^19^^,^^20^ Evidence on the efficacy, tolerability, and sustainability of low-dose supplementation remains scarce, despite its practical advantages for daily, long-term use.

To address this gap, the present randomized, double-blind, placebo-controlled trial investigated the effects of 12-week supplementation with low-dose (120 mg) and moderate-dose (204 mg) YBG among moderately stressed adults. The primary endpoint was the change in the Wisconsin Upper Respiratory Symptom Survey (WURSS-21) total severity score from baseline to week 12 (day 90). This score is calculated by summing items 1 through 20, which measure both symptom severity and functional impact. Secondary outcomes included URTI occurrence and assessments of fatigue, mood state, and health-related quality of life, while additional evaluations encompassed anthropometric measures, dietary intake records, and blood safety biomarkers. We hypothesized that daily low-to-moderate YBG supplementation would safely reduce URTI symptom severity and improve fatigue, mood, and overall quality of life without adverse effects on general health.

## Results

### Participant characteristics

Between January and November 2024, 198 moderately stressed adults were randomized, of whom 190 completed the study and were included in the intention-to-treat analysis: placebo (*n* = 65), YBG 120 mg (*n* = 62), and YBG 204 mg (*n* = 63). The baseline sociodemographic characteristics, including sex, race, marital status, educational level, employment status, and alcohol consumption, were comparable across groups (all *p* > 0.05). Anthropometric measures, including body weight, body mass index, body fat percentage, skeletal muscle mass, and baseline blood pressure, were also similar between groups.

A modest but statistically significant difference in age was observed at baseline (*p* = 0.038). Overall, participants had mean BMI values within the normal to overweight range (24.2 ± 5.5 kg/m^2^). No clinically relevant safety concerns were observed during the intervention period. The baseline characteristics are summarized in Table 1.

**Table 1:** Comparison of baseline sociodemographic and anthropometric between intervention and placebo groups [completers (*n* = 190)]

Characteristics	Placebo (*n* = 65)	YBG 120 mg (*n* = 62)	YBG 204 mg (*n* = 63)	Total (*n* = 190)	*p* value
**Sociodemographic Variables**					

Age, mean ± SD	27.85 ± 9.0	25.52 ± 6.7	29.35 ± 9.87	27.57 ± 8.7	∗0.038
Gender					
Male	23 (34.8)	19 (28.8)	25 (37.9)	67 (33.8)	0.536
Female	43 (65.2)	47 (71.2)	41 (62.1)	131 (66.2)	
Race, *n* (%)					
Malay	47 (71.2)	52 (78.8)	41 (62.1)	140 (70.7)	0.438
Non-Malays	19 (28.8)	14 (21.2)	25 (37.9)	58 (29.3)	
Marital Status, *n* (%)					
Single	48 (72.7)	55 (83.3)	45 (68.2)	148 (74.7)	0.164
Married/widow/divorce	18 (27.3)	11 (16.7)	21 (31.8)	50 (25.3)	
Educational Level, *n* (%)					
Certificate/diploma/bachelor	57 (32.4)	59 (33.5)	60 (34.1)	176 (88.9)	0.790
Masters/PhD	9 (40.9)	7 (31.8)	6 (27.3)	22 (11.1)	
Employment Status, *n* (%)					
Student	44 (32.6)	49 (36.3)	42 (31.1)	135 (68.2)	0.526
Employed	22 (34.9)	17 (12.6)	24 (38.1)	63 (31.8)	
Alcohol Status, *n* (%)					
Yes	7 (10.6)	10 (15.2)	6 (9.1)	23 (11.6)	0.532
No	59 (89.4)	56 (84.8)	60 (90.9)	175 (88.4)	
Weight (kg), mean ± SD	62.32 ± 15.35	64.24 ± 14.32	63.55 ± 13.62	63.37 ± 14.40	0.742
BMI (kg/m^2^), mean ± SD	24.06 ± 5.49	24.37 ± 5.34	24.23 ± 4.64	24.22 ± 5.15	0.943
Percentage body fat (%), mean ± SD	32.38 ± 8.83	32.92 ± 10.32	31.62 ± 9.92	32.31 ± 9.68	0.744
Skeletal muscle mass (kg), mean ± SD	22.45 ± 5.09	23.39 ± 5.60	23.85 ± 5.69	23.23 ± 5.47	0.326
Blood pressure systolic (mmHg), mean ± SD	109.08 ± 13.59	109.26 ± 13.07	111.55 ± 12.19	109.96 ± 12.95	0.474
Blood pressure systolic (mmHg), mean ± SD	71.17 ± 8.23	72.33 ± 9.16	73.62 ± 8.39	72.37 ± 8.62	0.263

**Dietary Intake (3 days food records)**					

Macronutrients					
Energy (kcal/day), mean ± SD	1,855 ± 574	1585 ± 525	1639 ± 522		∗0.013
Carbohydrate (g/day), mean ± SD	218.9 ± 73.9	183.5 ± 65.3	195.5 ± 70.5		∗0.016
Protein intake (g/day), mean ± SD	80.3 ± 30.3	68.3 ± 26.5	68.4 ± 26.1		∗0.021
Fat intake (g/day), mean ± SD	73.1 ± 25.2	64.3 ± 23.8	64.3 ± 23.1		0.061
Micronutrients					
Vitamin C (mg/day), mean ± SD	30.3 ± 20.9	31.0 ± 26.7	34.0 ± 23.2		0.639
Vitamin A (μg RE/day), mean ± SD	775.9 ± 428.4	871.2 ± 612.9	885.2 ± 598.0		0.474
Vitamin D (μg/day), mean ± SD	0.5 ± 1.0	0.5 ± 0.6	0.5 ± 0.9		0.908
Vitamin B6 (mg/day), mean ± SD	0.9 ± 0.6	0.9 ± 0.6	1.0 ± 0.5		0.844
Vitamin B12 (μg/day), mean ± SD	1.8 ± 1.3	1.6 ± 1.2	2.1 ± 2.0		0.281
Zinc intake (mg/day), mean ± SD	5.3 ± 3.6	5.1 ± 3.4	4.9 ± 3.0		0.763
Selenium intake (μg/day), mean ± SD	44.4 ± 22.9	44.3 ± 20.8	48.1 ± 26.6		0.582

Data were presented as *n* (%) or mean ± standard deviation (SD). Significant value at ∗*p* < 0.05 using the one-way ANOVA for continuous variables.

### URTI symptoms

After 12 weeks, both YBG groups showed lower mean WURSS-21 total severity scores than the placebo group (placebo = 14.09 ± 14.41; low-dose = 9.08 ± 13.29; moderate-dose = 8.81 ± 12.75; *p* = 0.05). The adjusted mean change from baseline was 3.42 ± 10.34 in placebo, −2.27 ± 10.27 in low-dose, and −3.51 ± 10.29 in moderate-dose YBG. Between-group comparisons demonstrated significant improvements in both intervention groups compared to the placebo group (*p* = 0.002 and *p* < 0.001, respectively).

The mean difference between placebo and moderate-dose YBG (−6.93) exceeded the minimum clinically important difference (MCID = 6.5) (45), indicating a clinically meaningful improvement. The proportion of responder participants achieving symptom reduction beyond the MCID was greater in both YBG groups (placebo = 13.85%, low-dose = 37.10%, moderate-dose = 41.27%; χ^2^ = 0.001), suggesting a dose-dependent effect (Table 2).

**Table 2:** Fatigue levels and WURSS-21 total severity and occurrence scores across intervention timepoints

Parameter	Placebo (*n* = 65)	YBG 120 mg (*n* = 62)	YBG 204 mg (*n* = 63)	Mean (95% CI)	Interaction effect			GLM (general repeated measures)					
								Time effect			Group effect		
					*p* value	Partial eta squared	Power	*p* value	Partial eta squared	Power	*p* value	Partial eta squared	Power
**General Fatigue**													

Baseline	10.11 ± 3.21	11.27 ± 3.43	10.54 ± 3.13	10.37 (10.07–10.68)	0.88	0.00	0.11	0.72	0.00	0.10	0.13	0.02	0.43
6 weeks	10.05 ± 3.54	10.68 ± 3.51	10.29 ± 3.51										
12 weeks	9.78 ± 3.04	10.56 ± 2.87	10.11 ± 2.70										

**Physical Fatigue**													

Baseline	9.63 ± 2.90	9.45 ± 2.87	9.90 ± 3.62	9.27 (8.96–9.57)	0.50	0.00	0.15	0.27	0.01	0.26	0.21	0.02	0.33
6 weeks	8.88 ± 3.20	9.18 ± 3.27	9.24 ± 3.61										
12 weeks	8.68 ± 3.55	9.05 ± 3.08	9.46 ± 3.09										

**Reduced Activity**													

Baseline	9.92 ± 2.91	10.37 ± 3.31	10.02 ± 3.19	9.80 (9.41–10.11)	0.39	0.01	0.31	0.54	0.00	0.14	0.55	0.01	0.15
6 weeks	9.40 ± 3.17	9.35 ± 2.74	10.27 ± 3.12										
12 weeks	9.52 ± 3.48	9.81 ± 3.31	9.56 ± 3.18										

**Reduced Motivation**													

Baseline	9.35 ± 2.65	9.90 ± 2.95	9.54 ± 3.02	9.24 (8.94–9.53)	0.62	0.01	0.20	0.73	0.00	0.10	0.72	0.00	0.10
6 weeks	9.03 ± 2.62	9.18 ± 2.85	9.52 ± 3.26										
12 weeks	8.94 ± 3.60	8.84 ± 2.84	8.83 ± 2.75										

**Mental Fatigue**													

Baseline	10.62 ± 3.23	10.68 ± 3.4	10.54 ± 2.29	10.17 (9.86–10.47)	0.72	0.01	0.15	0.26	0.01	0.26	0.83	0.00	0.08
6 weeks	10.23 ± 3.45	9.81 ± 2.78	10.05 ± 3.37										
12 weeks	10.03 ± 3.18	9.98 ± 2.96	9.56 ± 2.83										

**MFI Total Score**													

Baseline	48.37 ± 11.64	50.90 ± 13.32	49.54 ± 13.87	48.51 (47.21–49.82)	0.79	0.00	0.13	0.36	0.01	0.21	0.57	0.01	0.14
6 weeks	47.58 ± 13.79	48.19 ± 12.24	49.37 ± 14.16										
12 weeks	46.95 ± 14.51	48.24 ± 11.91	47.51 ± 12.32										

Parameter	Placebo	YBG 120 mg	YBG 204 mg	*p* value
WURSS-21 Total Severity Score throughout 12 weeks	14.09 ± 14.41	9.08 ± 13.29	8.81 ± 12.75	∗.05
Adjusted mean change	3.42 ± 10.34	−2.27 ± 10.27	−3.51 ± 10.29	<0.001

**WURSS-21 Symptom Occurrence throughout 12 weeks (%)**				

Group	1 or more episodes	No Episode	Total	0.09
Placebo	23 (35.4%)	42 (64.6%)	65	
YBG 120 mg	32 (51.6%)	30 (48.4%)	62	
YBG 204 mg	33 (52.4%)	30 (47.6%)	63	

Data are presented as mean ± SD. *N* = 190 participants (placebo *n* = 65; YBG 120 mg *n* = 62; YBG 204 mg *n* = 63). Statistical analysis: GLM repeated-measures ANOVA (time × group), adjusted for age and dietary energy, carbohydrate, and protein intake. The significance levels are indicated as follows: ∗*p* < 0.05, ∗∗*p* < 0.001, with comparisons made against the placebo group (post hoc pairwise comparisons using Bonferroni correction, if applicable). Note: The 204 mg dose achieved both statistical significance and clinical significance (exceeded MCID = 6.5), whereas the 120 mg dose achieved statistical significance but did not surpass the MCID threshold.

### Fatigue

A repeated-measures analysis of the Multidimensional Fatigue Inventory (MFI-20) indicated no significant group-by-time interactions for general, physical, or mental fatigue (all *p* > 0.05). Baseline total fatigue scores were relatively low across groups (placebo = 48.37, low-dose = 50.90, moderate-dose = 49.54 out of 100), indicating low-to-moderate fatigue levels well below clinical thresholds for severe fatigue.^21^ Although both YBG groups demonstrated small numerical reductions in fatigue from baseline to week 12 (placebo: −1.42; 120 mg: −2.66; 204 mg: −2.03), these changes were not statistically significant. The modest declines across all groups suggest a trend toward improved vitality but no differential treatment effect. Also, the low baseline scores may have contributed to a ceiling effect that limited the ability to detect further improvements (Table 2).

### Mood states

**Figure 1:**
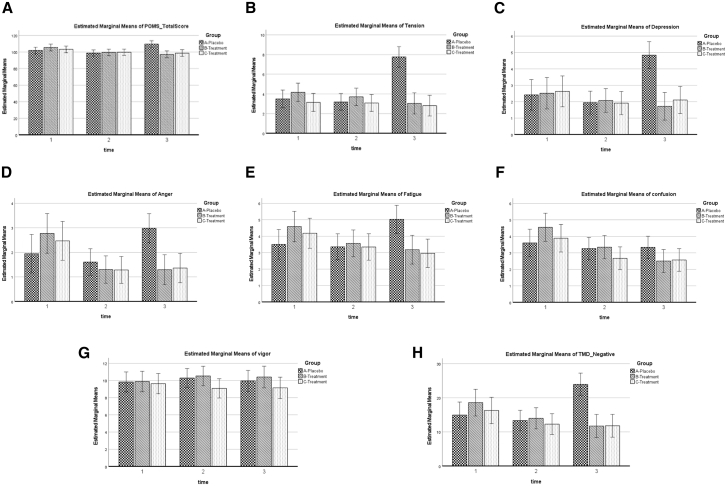
Mood state changes across treatment groups during the 12-week intervention (A) Total POMS score. (B) Tension. (C) Depression. (D) Anger. (E) Fatigue. (F) Confusion. (G) Vigor. (H) TMD (TMD_Negative). Three groups are shown: placebo (checkered bars), YBG 120 mg (diagonal striped bars), and YBG 204 mg (dotted bars). Measurements were taken at baseline (time 1), 6 weeks (time 2), and 12 weeks (time 3). Estimated marginal means were derived from a linear mixed-effects model adjusted for baseline dietary energy intake, carbohydrate intake, protein intake, and age. Error bars represent 95% confidence intervals. For (A–F) and (H), lower scores indicate reduced negative mood states; for (G) (Vigor), higher scores indicate increased energy and vitality.

Significant improvements were observed in negative mood domains, particularly tension, depression, anger, and total mood disturbance (TMD) scores at week 12 (Table 3). Baseline Profile of Mood States (POMS) total scores were comparable across groups. At week 12, both YBG groups showed reductions in total mood scores, whereas the placebo group exhibited an increase. Tension exhibited the most pronounced group-by-time effect, escalating in the placebo group while diminishing in both YBG groups (*p* < 0.001, η^2^*p* = 0.15). Depression and anger scores showed similar patterns, with reductions in the YBG groups and increases under placebo conditions (both *p* < 0.001). Fatigue also decreased in the YBG groups but increased in the placebo group (*p* < 0.001). Confusion scores declined modestly in the YBG groups, although the interaction did not reach statistical significance, while vigor remained unchanged across groups. Consistent with these domain-specific findings, TMD negative scores decreased in the YBG groups (120 mg: −6.52 ± 17.34; 204 mg: −4.38 ± 19.81) but increased in the placebo group (+8.55 ± 19.04; *p* < 0.001, η^2^*p* = 0.09) suggesting an overall enhancement in emotional well-being among supplemented participants (Figure 1).

**Table 3:** Mood state and quality of life status across intervention timepoints

Parameter	Placebo (*n* = 65)	YBG 120 mg (*n* = 62)	YBG 204 mg (*n* = 63)	Mean (95% CI)	Interaction effect			GLM (general repeated measures)					
								Time effect			Group effect		
					*p* value	Partial eta squared	Power	*p* value	Partial eta squared	Power	*p* value	Partial eta squared	Power
POMS Total Score													
Baseline	101.68 ± 15.01	106.60 ± 17.89	102.53 ± 16.55	101.63 (100.04–103.22)	0.00∗∗	0.07	0.98	0.19	0.01	0.32	0.29	0.01	0.27
6 weeks	98.71 ± 14.58	100.23 ± 15.67	99.38 ± 15.70										
12 weeks	109.17 ± 19.08	98.74 ± 14.91	97.83 ± 15.97										
Tension													
Baseline	3.52 ± 3.39	4.35 ± 4.20	2.90 ± 3.48	3.82 (3.44–4.20)	0.00∗∗	0.15	1.00	0.18	0.01	0.35	0.00∗∗	0.07	0.93
6 weeks	3.12 ± 3.45	3.87 ± 3.55	2.98 ± 3.46										
12 weeks	7.58 ± 5.48	3.32 ± 3.64	2.71 ± 3.18										
Depression													
Baseline	2.34 ± 3.55	2.74 ± 3.91	2.49 ± 3.89	2.46 (2.16–2.77)	0.00∗∗	0.07	0.98	0.05	0.02	0.58	0.03	0.04	0.68
6 weeks	1.88 ± 2.84	2.13 ± 2.63	1.94 ± 2.99										
12 weeks	4.71 ± 4.31	2.03 ± 2.58	1.94 ± 2.96										
Anger													
Baseline	1.92 ± 2.72	2.89 ± 3.41	2.38 ± 3.29	1.89 (1.63–2.16)	0.00∗∗	0.06	0.95	0.36	0.01	0.20	0.32	0.01	0.25
6 weeks	1.55 ± 2.55	1.34 ± 2.03	1.30 ± 1.96										
12 weeks	2.92 ± 2.72	1.42 ± 2.06	1.30 ± 2.36										
Fatigue													
Baseline	3.46 ± 3.12	4.79 ± 4.08	4.03 ± 3.75	3.75 (3.41–4.08)	0.00∗∗	0.05	0.92	0.21	0.01	0.31	0.54	0.01	0.15
6 weeks	3.31 ± 3.07	3.66 ± 3.12	3.30 ± 3.37										
12 weeks	4.92 ± 3.88	3.48 ± 3.47	2.78 ± 3.16										
Confusion													
Baseline	3.63 ± 3.17	4.69 ± 3.85	3.73 ± 3.06	3.31 (3.01–3.60)	0.13	0.02	0.49	0.73	0.00	0.08	0.47	0.01	0.18
6 weeks	3.17 ± 2.84	3.42 ± 2.83	2.70 ± 2.67										
12 weeks	3.29 ± 2.86	2.69 ± 2.77	2.43 ± 2.65										
Vigor													
Baseline	9.92 5.31	9.69 ± 4.50	9.73 ± 4.41	9.86 (9.40–10.32)	0.77	0.004	0.136	0.44	0.00	0.17	0.22	0.02	0.33
6 weeks	10.20± 4.08	10.42 ± 4.04	9.29 ± 5.22										
12 weeks	9.95 ± 4.94	10.19 ± 4.87	9.37 ± 5.10										
TMD Negative													
Baseline	14.88 ± 14.06	19.47 ± 16.84	15.54 ± 15.54	15.22 (13.86–16.59)	0.00∗∗	0.09	1.00	0.21	0.01	0.30	0.07	0.03	0.54
6 weeks	13.03 ± 12.56	14.42 ± 12.03	12.22 ± 12.20										
12 weeks	23.43 ± 15.93	12.95 ± 11.76	11.16 ± 12.92										

**SF-36**													

PF													
Baseline	89.23 ± 15.39	86.05 ± 21.25	82.14 ± 23.86	87.99 (86.01–89.98)	0.17	0.02	0.45	0.03∗	0.02	0.62	0.88	0.00	0.07
6 weeks	87.54 ± 23.03	87.26 ± 18.39	89.05 ± 18.62										
12 weeks	88.23 ± 21.18	90.24 ± 16.16	91.35 ± 12.39										
RLP													
Baseline	87.69 ± 24.66	81.85 ± 33.32	76.98 ± 34.87	85.80 (82.97–88.63)	0.16	0.02	0.49	0.30	0.01	0.26	0.84	0.00	0.08
6 weeks	86.92 ± 25.04	85.08 ± 29.84	77.49 ± 25.73										
12 weeks	87.31 ± 26.93	87.90 ± 24.27	89.68 ± 23.17										
Pain													
Baseline	81.04 ± 16.98	76.98 ± 18.74	73.06 ± 20.85	80.57 (78.94–82.20)	0.28	0.01	0.36	0.85	0.00	0.07	0.36	0.01	0.23
6 weeks	80.35 ± 17.51 ±	80.04 ± 17.83	81.43 ± 17.44										
12 weeks	84.31 ± 16.94	81.61 ± 17.47	86.11 ± 13.46										
GH													
Baseline	72.00 ± 15.86	68.71 ± 18.93	71.11 ± 16.47	72.66 (70.91–74.42)	0.75	0.00	0.13	0.98	0.00	0.05	0.24	0.02	0.31
6 weeks	74.15 ± 17.89	71.77 ± 17.39	70.00 ± 16.34										
12 weeks	77.38 ± 17.64	73.79 ± 15.41	75.16 ± 15.42										
RLE													
Baseline	66.67 ± 40.83	74.19 ± 38.37	67.72 ± 39.70	76.57 (72.95–80.18)	0.34	0.01	0.32	0.07	0.02	0.51	0.38	0.01	0.22
6 weeks	73.33 ± 38.28	86.56 ± 25.93	80.95 ± 34.24										
12 weeks	80.00 ± 34.26	77.96 ± 32.47	83.07 ± 32.17										
EF													
Baseline	59.38 ± 16.43	57.02 ± 18.32	59.84 ± 15.53	60.62 (58.91–62.32)	0.54	0.01	0.23	0.56	0.00	0.13	0.10	0.03	0.47
6 weeks	64.77 ± 17.69	59.92 ± 17.19	59.21 ± 17.72										
12 weeks	64.77 ± 18.32	61.05 ± 14.99	59.68 ± 19.03										
EW													
Baseline	69.11 ± 15.95	66.90 ± 18.25	70.67 ± 14.90	71.22 (69.56–72.88)	0.45	0.01	0.26	0.85	0.00	0.07	0.66	0.00	0.12
6 weeks	73.35 ± 17.24	72.84 ± 14.60	70.16 ± 14.95										
12 weeks	73.05 ± 17.71	73.48 ± 15.09	71.49 ± 14.98										
SF													
Baseline	71.92 ± 20.61	70.16 ± 25.44	68.85 ± 20.07	74.39 (72.24–76.53)	0.54	0.01	0.22	0.85	0.00	0.07	0.76	0.00	0.09
6 weeks	73.27 ± 22.41	74.60 ± 21.59	78.37 ± 18.54										
12 weeks	78.65 ± 23.67	75.20 ± 24.53	80.36 ± 17.47										
PHC													
Baseline	327.74 ± 51.69	313.59 ± 64.96	303.29 ± 70.87	326.45 (320.16–332.74)	0.28	0.01	0.35	0.24	0.01	0.28	0.052	0.01	0.16
6 weeks	328.96 ± 61.68	324.15 ± 59.48	327.94 ± 56.12										
12 weeks	337.85 ± 68.41	333.55 ± 50.39	342.62 ± 43.03										
MHC													
Baseline	267.08 ± 71.45	268.27 ± 83.86	267.08 ± 69.34	282.35 (274.95–289.76)	0.88	0.00	0.09	0.38	0.01	0.18	0.92	0.00	0.06
6 weeks	285.88 ± 72.52	293.91 ± 48.64	288.15 ± 66.93										
12 weeks	296.47 ± 80.36	287.69 ± 65.61	294.66 ± 70.85										
Total SF-36													
Baseline	593.69 ± 105.99	581.86 ± 134.60	570.38 ± 125.09	608.39 (595.59–621.19)	0.77	0.00	0.12	0.33	0.01	0.21	0.83	0.00	0.08
6 weeks	615.51 ± 122.80	617.22 ± 94.81	615.06 ± 110.70										
12 weeks	633.59 ± 136.37	621.24 ± 102.86	637.09 ± 102.75										

Data are presented as mean ± SD. *N* = 190 participants (placebo *n* = 65; YBG 120 mg *n* = 62; YBG 204 mg *n* = 63). Statistical analysis: GLM repeated-measures ANOVA (time × group), adjusted for age and dietary energy, carbohydrate, and protein intake. The significance levels are indicated as follows: ∗*p* < 0.05, ∗∗*p* < 0.001, with comparisons made against the placebo group (post hoc pairwise comparisons using Bonferroni correction, if applicable). Abbreviations: SF-36, 36-item short form health survey; RLP, role limitations due to physical health; Pain, bodily pain; GH, general health; RLE, role limitations due to emotional problems; EF, emotional functioning; EW, energy/vitality; SF, social functioning; MHC, mental health composite.

### Quality of life

Improvements in the Short Form Health Survey (SF-36) subdomains were most evident in physical functioning (PF), bodily pain, and physical health composite (PHC) scores after 12 weeks. The moderate-dose YBG group showed the largest numerical gains in bodily pain (Δ = +13.06 ± 23.68, *p* = 0.05) and PHC (Δ = +39.33 ± 75.00, *p* = 0.08), approaching statistical significance. No significant changes were noted for mental health composite (MHC) or emotional role limitations (*p* > 0.05) (Table 3). These findings suggest modest yet consistent improvements in perceived physical quality of life with YBG supplementation. However, given the exploratory nature and insufficient power for detecting small effects on secondary outcomes, these trends warrant further investigation in larger, adequately powered studies.

### Biochemical safety profile

**Figure 2:**
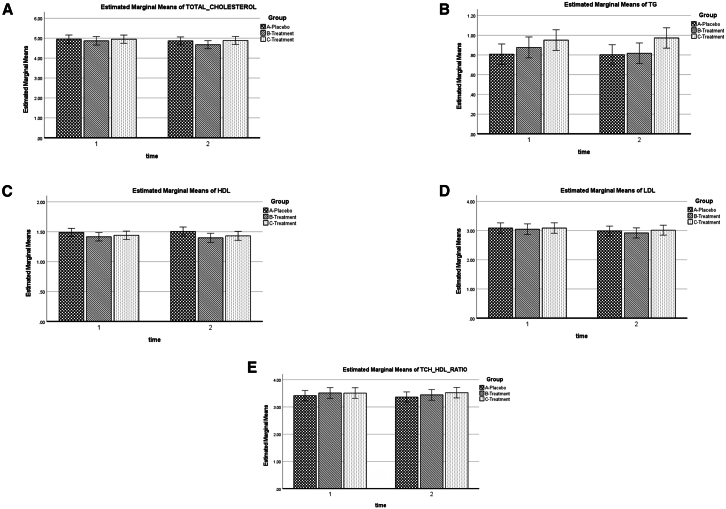
Lipid profile biomarkers across treatment groups during the 12-week intervention (A) Total cholesterol. (B) Triglycerides (TG). (C) High-density lipoprotein cholesterol (HDL). (D) Low-density lipoprotein cholesterol (LDL). (E) Total cholesterol/HDL ratio (TC/HDL). Three groups are shown: placebo (checkered bars), YBG 120 mg (diagonal striped bars), and YBG 204 mg (dotted bars). Measurements were taken at baseline (time 1) and week 12 (time 2). Estimated marginal means were derived from a linear mixed-effects model adjusted for baseline dietary energy intake, carbohydrate intake, protein intake, and age. Error bars represent 95% confidence intervals.

**Figure 3:**
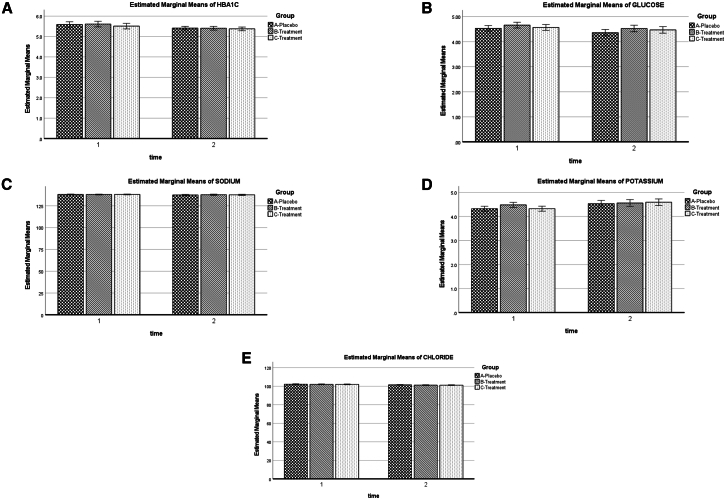
Glycemic and electrolyte biomarkers across treatment groups during the 12-week intervention (A) Hemoglobin A1c (HbA1c). (B) Glucose. (C) Sodium. (D) Potassium. (E) Chloride. Three groups are shown: placebo (checkered bars), YBG 120 mg (diagonal striped bars), and YBG 204 mg (dotted bars). Measurements were taken at baseline (time 1) and week 12 (time 2). Estimated marginal means were derived from a linear mixed-effects model adjusted for baseline dietary energy intake, carbohydrate intake, protein intake, and age. Error bars represent 95% confidence intervals. All biomarkers remained within normal physiological ranges.

**Figure 4:**
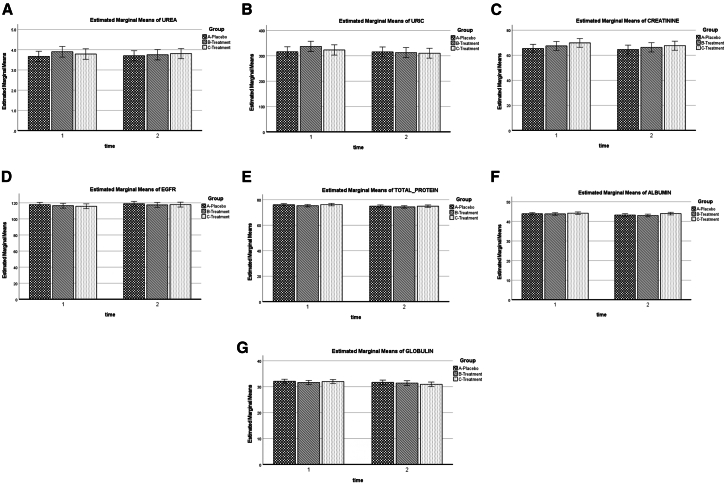
Renal function and protein biomarkers across treatment groups during the 12-week intervention (A) Urea. (B) Uric acid. (C) Creatinine. (D) Estimated glomerular filtration rate (eGFR). (E) Total protein. (F) Albumin. (G) Globulin. Three groups are shown: placebo (checkered bars), YBG 120 mg (diagonal striped bars), and YBG 204 mg (dotted bars). Measurements were taken at baseline (time 1) and week 12 (time 2). Estimated marginal means were derived from a linear mixed-effects model adjusted for baseline dietary energy intake, carbohydrate intake, protein intake, and age. Error bars represent 95% confidence intervals. All biomarkers remained within normal physiological ranges throughout the study.

No adverse biochemical trends were detected. Total protein, albumin, creatinine, liver enzymes, and lipid profiles remained within their respective reference ranges across all groups throughout the trial (Table 4). The longitudinal trajectory of all measured safety biomarkers, presented as estimated marginal means, is shown in Figures 2, 3, and 4. A small but significant reduction in serum uric acid was observed in both YBG groups compared with the placebo group (*p* = 0.02), although all values remained within the normal reference range. No significant group-by-time effects were observed for glucose, HbA1c, or lipid fractions (*p* > 0.05), indicating that low-to-moderate YBG doses did not adversely affect metabolic parameters.

**Table 4:** Effect of intervention interaction on biochemical parameters between YBG groups and placebo from baseline to week 12 (mean ± SD)

	Normal range	Placebo (*n* = 65)	YBG 120 mg (*n* = 63)	YBG 204 mg (*n* = 62)	Group x Time effect		
					*p* value	Partial eta squared	Observed power
Total Cholesterol (mmol/L)							
Baseline	<5.2	4.95 ± 0.91	4.78 ± 0.76	5.03 ± 0.97	0.37	0.01	0.22
Week 12		4.85 ± 0.86	4.61 ± 0.72	4.94 ± 0.88			
TG (mmol/L)							
Baseline	<1.68	0.82 ± 0.41	0.84 ± 0.40	0.99 ± 0.52	0.34	0.01	0.24
Week 12		0.81 ± 0.36	0.78 ± 0.39	0.97 ± 0.50			
HDL (mmol/L)							
Baseline	>1.03	1.48 ± 0.27	1.42 ± 0.30	1.45 ± 0.26	0.58	0.01	0.14
Week 12		1.49 ± 0.32	1.42 ± 0.31	1.43 ± 0.28			
LDL (mmol/L)							
Baseline	<2.58	3.09 ± 0.80	2.97 ± 0.59	3.15 ± 0.81	0.78	0.00	0.09
Week 12		2.99 ± 0.73	2.86 ± 0.57	3.07 ± 0.81			
TCH: HDL Ratio							
Baseline	<5.0	3.44 ± 0.82	3.44 ± 0.78	3.55 ± 0.78	0.44	0.10	0.19
Week 12		3.39 ± 0.88	3.36 ± 0.74	3.52 ± 0.84			
HbA1C %							
Baseline	<6.5	5.58 ± 0.75	5.58 ± 0.52	5.55 ± 0.39	0.65	0.01	0.12
Week 12		5.41 ± 0.33	5.39 ± 0.46	5.39 ± 0.32			
Glucose (mmol/L)							
Baseline	4.1–6.1	4.53 ± 0.31	4.66 ± 0.59	4.58 ± 0.39	0.72	0.00	0.10
Week 12		4.36 ± 0.39	4.51 ± 0.66	4.49 ± 0.48			
Sodium (mmol/L)							
Baseline	136–146	138.09 ± 1.89	137.85 ± 1.71	137.98 ± 1.72	0.79	0.00	0.09
Week 12		137.34 ± 1.52	137.82 ± 2.40	137.52 ± 3.60			
Potassium (mmol/L)							
Baseline	3.5–5.1	4.32 ± 0.42	4.47 ± 0.45	4.38 ± 0.39	0.22	0.02	0.32
Week 12		4.55 ± 0.51	4.54 ± 0.57	4.61 ± 0.58			
Chloride (mmol/L)							
Baseline	101–109	102.14 ± 1.99	102.03 ± 1.89	102.05 ± 1.50	0.84	0.00	0.08
Week 12		101.49 ± 1.38	101.24 ± 1.33	101.19 ± 1.11			
Urea (mmol/L)							
Baseline	2.5–8.0	3.68 ± 0.91	3.87 ± 1.08	3.80 ± 1.09	0.56	0.01	0.15
Week 12		3.73 ± 1.03	3.75 ± 1.00	3.78 ± 1.05			
Uric Acid (umol/L)							
Baseline	155–357	320.62 ± 82.55	333.50 ± 90.76	321.79 ± 78.29	0.04∗	0.03	0.60
Week 12		320.92 ± 86.73	307.81 ± 85.25	309.56 ± 76.47			
Creatinine (umol/L)							
Baseline	45–84	66.54 ± 14.94	66.87 ± 14.70	69.16 ± 14.63	0.52	0.01	0.16
Week 12		65.42 ± 14.56	65.82 ± 15.49	67.13 ± 14.67			
eGFR (mL/min/1.73 m²), > 60 mL/min/1.73 m²							
Baseline	90+	116.89 ± 14.93	118.56 ± 13.73	114.46 ± 12.39	0.76	0.00	0.09
Week 12		118.43 ± 93.97	119.31 ± 14.49	116.38 ± 12.70			
Total Protein (g/L)							
Baseline	66–83	76.08 ± 4.15	75.37 ± 3.41	76.10 ± 3.42	0.91	0.00	0.06
Week 12		75.08 ± 4.07	74.40 ± 3.32	74.81 ± 3.84			
Albumin (g/L)							
Baseline	32–52	43.92 ± 2.75	43.94 ± 2.34	44.03 ± 2.73	0.43	0.10	0.19
Week 12		43.28 ± 2.99	43.32 ± 3.00	43.65 ± 3.05			
Globulin (g/L)							
Baseline	21–40	32.15 ± 3.42	31.58 ± 2.95	32.06 ± 2.91	0.33	0.01	0.25
Week 12		31.80 ± 4.17	31.15 ± 3.36	31.13 ± 3.56			

Values are presented as mean ± standard deviation (SD). *N* = 190 participants (placebo *n* = 65; YBG 120 mg *n* = 62; YBG 204 mg *n* = 63). Statistical analysis: GLM repeated-measures ANOVA (time × group), adjusted for age and dietary energy, carbohydrate, and protein intake. The significance levels are indicated as follows: ∗*p* < 0.05, ∗∗*p* < 0.001, with comparisons made against the placebo group. Abbreviations: TG, triglycerides; HDL, high-density lipoprotein cholesterol; LDL, low-density lipoprotein cholesterol; TCH, total cholesterol; HbA1c, glycated hemoglobin; eGFR, estimated glomerular filtration rate.

### Adverse events and safety outcomes

No serious adverse events (SAEs) occurred during the 12-week intervention, and all 190 participants who completed the study reported no treatment-related adverse events. Minor, transient symptoms such as mild gastrointestinal discomfort and headaches were noted across all groups; however, these events were short-lived and assessed as unlikely related to the study supplement. No participants withdrew due to adverse events. All discontinuations were attributed to unrelated health issues, confirmed pregnancy, personal reasons, loss to follow-up, travel, and relocation.

## Discussion

### Study rationale and principal findings

This randomized, double-blind, placebo-controlled trial evaluates the efficacy and safety of low-dose YBG (1,3/1,6) supplementation (120 mg and 204 mg/day) in moderately stressed adults. The term ‘moderate-stress adults’ in this study refers to individuals with perceived stress scores ranging from 14 to 26, representing those who experience ongoing psychological or occupational stress typical of modern life, yet remain within a non-clinical range. This population represents functionally healthy individuals who experience stress-related immune and mood disturbances, distinct from both low-stress individuals in the general population and those with diagnosed psychological disorders. To date, most clinical investigations have focused on higher doses (typically 250–500 mg/day),^16^ which have consistently demonstrated immunomodulatory effects and clinical benefits in reducing the URTIs severity and improving host immune readiness. However, whether comparable physiological responses can be achieved at lower, more practical intake levels remains insufficiently explored.

Our results demonstrate that both low-dose regimens significantly reduce URTI symptom severity compared with placebo, with the 204 mg group achieving the most significant and clinically meaningful reduction (mean adjusted change = −3.51 ± 10.29, *p* < 0.001). Participants receiving YBG also reported lower frequencies of symptomatic episodes and improvements in negative mood domains (tension, depression, and anger) measured by POMS-40, while fatigue and quality of life indices remained unchanged. These findings collectively support the hypothesis that even physiologically modest doses of YBG can enhance immune resilience and psychological well-being without adverse metabolic consequences. Importantly, our results align with previous human intervention studies, which shows that beta-1,3/1,6-glucans from yeast reduce symptom severity of URTIs while improving mood states in moderately to highly stressed individuals. For example, Talbott et al.^22^ reported reduced URTI symptoms and improved mood following YBG supplementation in stressed adults.^22^ The findings contribute to existing knowledge by suggesting a potential threshold of biological responsiveness at lower supplementation levels, which may help inform future formulation considerations and practical application.

### Mechanistic interpretation and dose relevance

Recent immunobiological evidence suggests that YBG, even at lower doses, primes innate immune pathways through pattern recognition receptors such as dectin-1 and complement receptor 3 (CR3). This receptor-mediated training enhances monocyte and macrophage responsiveness,^23^ facilitating rapid pathogen clearance upon subsequent exposure. Our findings align with this mechanism, where lower URTI symptom severity and fewer infection episodes reflect improved mucosal and systemic immune vigilance rather than pharmacological immune activation.^15^^,^^24^^,^^25^ This concept parallels findings from other functional bioactive, such as probiotics and prebiotics, which induce measurable improvements in immune and metabolic outcomes at low, physiologically relevant doses.^26^ Thus, the current results extend prior high-dose YBG evidence by demonstrating similar beneficial effects at substantially lower intakes, broadening its practical applicability for preventive health strategies.

### Population characteristics and confounding control

The participant population consisting of adults with moderate stress represents a functionally healthy but stress-vulnerable group prone to immune and mood perturbations. This selection bridges the gap between healthy individuals and those with psychological disorders, targeting a realistic preventive-health demographic. The baseline characteristics, including sex distribution, age, and stress levels, were balanced across study arms, and potential confounders were statistically adjusted using a general linear model repeated-measures (GLM). Specifically, age and dietary energy, carbohydrate, and protein intake were included as covariates due to significant baseline differences (*p* < 0.05) and their known influence on immune function, inflammatory status, and psychophysiological outcomes.^27^ The selection of these macronutrients is based on their biological significance, as energy availability affects immune cell metabolism and fatigue, carbohydrates affect glucose availability for immune function and gut microbiota composition, and protein is crucial for antibody synthesis and immune cell proliferation.^28^ Fat intake was excluded as a covariate due to its lack of significant variation at baseline (*p* > 0.05) and its minimal direct short-term impact on the key immunological and psychophysiological outcomes evaluated in this investigation. The stability of blood metabolic markers (urea, creatinine, and albumin) after the intervention indicated that participants sustained a constant metabolic status, hence supporting the appropriateness of the statistical adjustments.

### Comparison with prior YBG evidence and placebo-controlled interpretation

Our findings on low-dose supplementation are directionally consistent with prior human trials employing higher YBG intakes (250–1,000 mg/day),^17^^,^^29^^,^^30^ which reported decreased URTI incidence and improved psychological outcomes. In our cohort, both the 120 mg and 204 mg groups demonstrated lower WURSS-21 total severity and a higher proportion of episode-free participants, supporting the reproducibility of YBG’s respiratory benefits across dose ranges. Mood improvements observed across several POMS domains further substantiate psychoneuroimmunological links previously proposed for YBG, wherein reduced inflammatory signaling and enhanced immune efficiency alleviate negative affective states.^31^

Mechanistically, orally administered YBG acts primarily through localized mucosal immune activation instead of systemic absorption. Upon ingestion, YBG particles are captured by Peyer’s patches and M cells, phagocytosed by intestinal macrophages, and conveyed to lymphoid organs including the spleen, liver, and bone marrow, where they initiate immune cascades despite minimal systemic bioavailability (<5%), as demonstrated by Samuelsen et al.^15^ The authors reported that these macrophage-mediated pathways enhance secretory IgA and IL-10 production, strengthen antiviral defense, and fine-tune inflammatory responses, thereby providing a physiological explanation for the observed reductions in URTI symptoms and improved psychological resilience across both high and low-dose YBG studies. Moreover, gut-mediated immune signaling may also affect neuroimmune interaction, contributing to the enhanced mood and emotional well-being observed in participants supplemented with YBG.

The inclusion of a placebo arm allowed differentiation between intervention effects and natural variability inherent to moderately stressed adults. Within-group comparisons showed that minor symptom fluctuations occurred across all groups, reflecting the normal course of upper respiratory and psychological responses over a 12-week period. However, only the YBG groups exhibited consistent directional improvements beyond these background variations, with reductions in URTI symptom severity and mood disturbances exceeding changes observed in the placebo group. These findings suggest that the observed benefits are unlikely to be attributed solely to regression to the mean or seasonal variation but rather to the physiological influence of YBG supplementation. The stability of most biochemical parameters across all groups further supports that the detected changes were specific, controlled, and not confounded by random baseline shifts.^22^

In contrast, fatigue (measured by MFI-20) and quality of life (SF-36) scores did not differ significantly between groups. This divergence from certain high-dose or athlete-based trials likely reflects our cohort’s relatively low baseline fatigue and the multifaceted nature of this construct, which is shaped by factors such as sleep,^32^^,^^33^ circadian rhythm,^34^ physical activity,^35^ and psychosocial stressors. These factors, often stable over a 12-week period, may not be fully modifiable through immunonutrition alone. Consequently, the lack of change in fatigue levels suggests that YBG may exert a targeted rather than broad effect spectrum effects on psychophysiological outcomes.

### Clinical interpretation of biochemical findings

Additionally, the statistically significant reduction in serum uric acid at 204 mg/day is an unexpected yet biologically plausible observation. Although absolute values remained within the normal range. The clinical significance of this finding is unclear, as the study was not designed or powered to detect changes in biochemical markers. This observed reduction should be interpreted as an exploratory finding. Other biochemical indices remained unchanged, reinforcing the supplement’s metabolic neutrality and favorable safety profile. Future studies specifically designed to investigate metabolic and biochemical effects of YBG supplementation are needed to determine whether this finding is reproducible and clinically meaningful.

### Primary outcome measurement validity

The Malay WURSS-21 validation manuscript is presently undergoing peer review. The issue is significantly alleviated by our bilingual questionnaire administration method where all participants received both Malay and English versions of the WURSS-21 and could select their preferred language or cross-reference across versions for clarification. Considering that the English WURSS-21 offers substantial psychometric features and notable international validation (Barrett et al., 2005),^36^ this bilingual methodology ensured that the assessment was not exclusively dependent on an unvalidated translation. Initial validation data exhibit exceptional psychometric attributes (Cronbach’s alpha = 0.92, Kaiser-Meyer-Olkin (KMO) = 0.862, four-factor structure, and strong concurrent validity), suggesting negligible random measurement error. Furthermore, all intervention groups and the placebo adhered to the identical instrument delivery technique, indicating that any systematic measurement bias would uniformly impact all groups, hence not biasing between-group comparisons. The reported effect sizes (mean difference of −6.93 for moderate-dose YBG compared to placebo) above the minimal clinically important difference (MCID = 6.5) by a significant margin, instilling confidence that the findings are not solely due to measurement error.

### Clinical significance vs. statistical significance

The analysis of the primary endpoint (WURSS-21 total severity at week 12) revealed adjusted mean reductions from placebo of −5.69 points (95% CI: −9.2 to −2.2) for the 120 mg group and −6.93 points (95% CI: −10.4 to −3.5) for the 204 mg group, both achieving statistical significance (120 mg: *p* = 0.002; 204 mg: *p* < 0.001). However, only the 204 mg dose exceeded the established minimal clinically important difference (MCID = 6.5 points), whereas the 120 mg dose, despite its statistical significance, remained slightly below this threshold. Responder analysis further supported this dose-dependent pattern: 41.27% of participants administered 204 mg and 37.10% receiving 120 mg achieved clinically meaningful reductions (≥6.5 points), in contrast to 13.85% in the placebo arm (χ^2^ = 14.2, *p* = 0.001).

The results highlight a nuanced dose-response relationship with important clinical implications. The 120 mg dose demonstrated biological activity as evidenced by its significant between-group difference relative to placebo and its nearly 3-fold higher responder rate, yet the mean symptom reduction did not attain the magnitude required for population-level clinical meaningfulness. This suggests that while many individuals benefit substantially from the lower dose, the average group effect remains modest and below the MCID threshold. Such inter-individual variability raises the possibility that certain biological or psychosocial characteristics (e.g., baseline immune status, genetic variation, or microbiome composition) may influence responsiveness to lower-dose supplementation, warranting further investigation in mechanistic studies.^37^ In contrast, the 204 mg dose showed consistent effectiveness across both population-level and individual-level metrics, surpassing the MCID and producing the highest proportion of clinically meaningful responders. The clearer clinical impact at this higher dose aligns with a plausible biological gradient, whereby greater availability of beta-glucan may more reliably engage pattern recognition receptors such as dectin-1 and CR3, leading to more substantial regulation of innate immune pathways and mucosal defense.^38^

Collectively, these findings highlight the distinction between statistical significance and clinical meaningfulness. While 120 mg offers measurable and statistically significant improvements, 204 mg appears as the more dependable regimen for achieving clinically relevant symptom reductions in moderately stressed adults. This dose-stratified efficacy pattern has practical implications for supplement formulation, public health recommendations, and future trial design, establishing 204 mg as the minimally effective dose for consistent clinical benefit while acknowledging that lower doses may still benefit selected individuals.

### Mood outcomes and psychobehavioral context

Across the various domains of the POMS (tension, depression, and anger), both the low-dose and moderate-dose groups demonstrated statistically significant reductions compared with the placebo group, and a lower total mood-disturbance score at 12 weeks. While these mood improvements achieved statistical significance, their absolute magnitude was modest. It should therefore and therefore be interpreted as enhancements in psychological well-being rather than as indications of a clinically significant intervention for affective disorders. This interpretation is consistent with our enrollment of a moderately stressed (but non-clinical) adult cohort, and aligns with the trial’s positioning of mood as a secondary outcome. These findings also align with current evidence that supplementation with YBGs sources may favorably influence mood and neuro-behavioral domains via immuno-metabolic or gut-brain-axis pathways.^21^ For example, a recent systematic review found that YBGs appear to reduce self-reported fatigue in clinical and sub-clinical populations, albeit with heterogeneity across studies.^17^ Similarly, in a healthy-adult dietary-fiber intervention involving young women, higher beta-glucan intake from barley was associated with improved mental-health and health-related quality of life (HR-QoL) scores over 4 weeks.^39^ Together, these data support the notion that modest YBG supplementation may enhance mood outcomes in non-clinical “stress-elevated” populations, but are consistent with our finding of moderate effect sizes and the need for cautious interpretation with respect to formal mood-disorder claims.

### Null findings for fatigue and quality of life

No statistically significant between-group differences in fatigue (assessed via MFI-20) or health-related quality of life (via SF-36) were observed following the 12-week intervention. These null findings are best interpreted in light of three salient factors including the baseline characteristics, study design constraints, and instrument responsiveness. At enrollment, participants reported low-to-moderate baseline fatigue (mean MFI-20-49/100)^21^ and generally preserved SF-36 scores, resulting in limited scope for measurable improvement and creating potential ceiling effects. The trial was powered adequately for detecting changes in the primary endpoint, URTI symptom severity, but not for the smaller effect sizes anticipated for these secondary participant-reported outcomes. Consequently, the modest within-group reductions in MFI-20 scores (placebo: −1.42; 120 mg: −2.66; 204 mg: −2.03), while consistent in direction, were likely below the threshold of reliable detection in the current sample size. Moreover, fatigue in moderately stressed, otherwise healthy adults is frequently multifactorial and relatively stable, which may restrict responsiveness to a singular immunomodulatory intervention.

Although validated, the MFI-20 and SF-36 may possess restricted sensitivity to detect subtle, clinically meaningful changes in high-functioning populations. Together, these considerations suggest that the absence of statistical significance reflects methodological and contextual limitations, including limited baseline impairment and insufficient power, rather than robust evidence of no effect. Future investigations should target populations with elevated baseline fatigue burden, employ larger samples powered specifically for these endpoints, and explore longer intervention durations or escalated dosing to more definitively evaluate potential benefits for fatigue and quality of life.

### Broader implications

These findings position YBG as a practical adjunct within the immunonutrition paradigm. Demonstrating efficacy at a moderate dose of 204 mg helps address adherence and affordability considerations often associated with higher doses, supporting YBG’s potential suitability for longer-term use in general populations. The observed improvements in negative mood states suggest that YBG may have a modest influence on immune-neural interactions, particularly in stress-exposed yet otherwise healthy adults. Collectively, these outcomes suggest a balanced role for YBG in supporting both immune and psychological well-being, warranting further investigation in larger and more diverse cohorts.^38^^,^^40^^,^^41^

Future investigations should integrate objective biomarkers of immune activation (e.g., cytokine panels and salivary IgA) and microbiota composition to clarify the mechanistic pathways linking immune priming and emotional regulation. Longer-term studies across diverse populations will determine the durability and generalizability of these effects.

In conclusion, this randomized, double-blind, placebo-controlled trial demonstrated that low-to-moderate doses of YBG supplementation alleviated upper respiratory symptom severity and enhanced mood in moderately stressed adults over a 12-week period. Significantly, although both doses (120 mg and 204 mg) exhibited statistical efficacy, only the 204 mg dose attained clinically meaningful improvement, thereby establishing it as the minimally effective dose for practical use. These findings indicate that YBG has a supporting role as an immunonutrient in maintaining respiratory and psychological health. However, given the limited duration and specific population studied, these results should be considered preliminary. A comprehensive, extended, and more diverse clinical studies are needed to confirm these outcomes, validate optimal dosing, and elucidate the mechanisms underlying YBG’s immunomodulatory effects.

### Limitations of the study

While natural variability in respiratory symptoms, mood, and fatigue is expected in this population, the inclusion of a placebo group provided an essential benchmark to distinguish true intervention effects from background fluctuations. The study was powered *a priori* using the Chan and Chan (2003)^42^ randomized controlled trial formula, with reference parameters derived from a prior YBG trial in marathon runners (Mah et al., 2020).^14^ Although that population differs from moderately stressed adults, it offered the closest available immune challenge comparator. As a result, the sample size was designed to detect clinically meaningful changes in the primary endpoint (URTI symptom severity) but not small to moderate shifts in secondary outcomes or biochemical variables, which were exploratory.

The study lacked sufficient power to identify subtle effects on secondary outcomes, including fatigue or quality of life. Additionally, the functionally healthy population with minimal baseline impairment likely produced ceiling effects that constrained the potential for measurable improvement. These null findings should therefore be interpreted as inconclusive rather than definitively negative. The observed reduction in serum uric acid should similarly be viewed as a preliminary metabolic signal rather than a definitive physiological change, given that the study was not designed for biomarker-focused endpoints. Larger, biomarker-specific trials will be needed to validate these findings.

It is important to emphasize that this study reports biological efficacy under controlled trial conditions, not real-world effectiveness. Therefore, the results may not generalize to populations with lower adherence or less structured support than the highly motivated participants in this trial. This contextualizes the use of an exclusive per-protocol analysis, which, although justified by the minimal and balanced dropout rate (4%), inherently limits generalizability (Table S1). Nevertheless, the 96% retention rate indicates that YBG supplementation was highly acceptable and feasible in moderately stressed adults within a research setting.

All questionnaires (WURSS-21, POMS-40, MFI-20, and SF-36) were provided in both Malay and English, allowing participants to use their preferred language or cross-reference between versions for clarity, a key consideration for the primary outcome. Although the Malay WURSS-21 validation study demonstrated excellent psychometric properties (Cronbach’s alpha = 0.92, KMO = 0.862), the validation manuscript remains under peer review and results warrant confirmation once the validation manuscript is formally published. The concurrent availability of the established English version (Barrett et al., 2005)^36^ ensured measurement quality was not dependent solely on an unpublished adaptation.

Lastly, the study relied on validated self-report instruments to assess clinical and psychological outcomes. Despite their widespread use, self-reported measures are intrinsically subjective and susceptible to the influence of daily stressors, recall bias, or individual interpretation. To complement subjective reports and strengthen mechanistic interpretation, future research should incorporate objective or biomarker-based indices, such as inflammatory cytokines, salivary IgA, or wearable-derived fatigue measures.

## Resource availability

### Lead contact

Further information and requests for resources should be directed to the lead contact, Professor Suzana Shahar (suzana.shahar@ukm.edu.my).

### Materials availability

This study used Baker’s yeast commercially available from Wellmune YBG 1,3/1,6 (*Saccharomyces cerevisiae*; supplied by Aqurate Ingredients International Sdn. Bhd., Malaysia). No new unique materials were generated. Requests for study materials should be directed to the lead contact.

### Data and code availability

• Processed data supporting the findings of this study have been deposited in Mendeley. Data are publicly accessible at: https://data.mendeley.com/datasets/vtfm6hb9wn/1
• This paper does not report original code.
• Any additional information required to reanalyze the data reported in this paper is available from the lead contact upon request.

## Acknowledgments

We thank the staff of the Centre for Healthy Ageing and Wellness (H-Care), Faculty of Health Sciences, 10.13039/501100004515Universiti Kebangsaan Malaysia, for their assistance in the preparation of the study protocol and trial coordination. This study was supported by an educational grant from Mead Johnson Nutrition Sdn. Bhd., Malaysia (grant no. NN-2022-027). The trial was conducted at the Centre for Healthy Ageing and Wellness (H-Care), Faculty of Health Sciences, 10.13039/501100004515Universiti Kebangsaan Malaysia.

## Author contributions

All authors made substantial contributions in the process of critical revision of the manuscript for optimum intellectual content. N.N.M.H. drafted the manuscript under the supervision of S.S.; N.N.M.H., S.S., M.I., H.F.M.R., N.I., S.G.H.T., M.F.A.H., K.R. and T.S. were involved in designing the study; N.N.M.H. was responsible for conducting the data collection; for data analysis using SPSS software, S.S., H.F.M.R., M.I., N.I., S.G.H.T., M.F.A.H. and K.R. guided N.N.M.H. with their knowledge and experience in performing statistical analysis; S.S. is responsible for the overall content as the guarantor. All authors have read, edited and approved the final draft of this manuscript.

## Declaration of interests

The authors have no conflicts of interest to declare.

## STAR★Methods

### Key resources table

**Table:** 

REAGENT or RESOURCE	SOURCE	IDENTIFIER
**Deposited data**		

Processed clinical trial data	Mendeley Data	https://data.mendeley.com/datasets/vtfm6hb9wn/1

**Experimental models: Organisms/strains**		

Baker’s yeast beta-glucan 1,3/1,6	Wellmune® Kerry Group	CAS: 9051-97-2

**Software and algorithms**		

IBM SPSS Statistics 29	IBM Corporation	https://www.ibm.com/spss
GraphPad Prism	GraphPad Software	https://www.graphpad.com
Nutritionist Pro™	Axxya Systems	https://www.nutritionistpro.com

**Other**		

WURSS-21 questionnaire	Barrett et al., 2005	https://doi.org/10.1016/j.jaci.2005.01.058
POMS-40 questionnaire	McNair et al., 1971	–
MFI-20 questionnaire	Smets et al., 1995	–
SF-36 questionnaire	Ware & Sherbourne	–
TANITA SC-330 body composition analyzer	TANITA Corporation	Model SC-330
Electronic blood pressure machine	Omron Kyoto, Japan	Model HEM-8712

### Experimental model and study participant details

#### Study design and ethical approval

A 12-week, randomized, double-blind, placebo-controlled, parallel-group clinical trial was conducted to evaluate the efficacy of yeast beta-glucan (YBG 1,3/1,6) supplementation among moderately stressed in Malaysian adults. Ethical approval was obtained from the Universiti Kebangsaan Malaysia Research Ethics Committee (UKM/PPI/111/8/JEP-2023-211). The study was registered with ISRCTN 48336189 and adhered to the Declaration of Helsinki and SPIRIT guidelines.

#### Participants characteristics

A total of 198 adults aged 18–59 years with moderate stress (Perceived Stress Scale-10 score 14-26; PHQ-9 ≤ 9) and ≥1 URTI episode within 6 months were recruited from Klang Valley, Malaysia. Recruitment was conducted at Hospital Canselor Tuanku Muhriz and the Centre for Healthy Ageing & Wellness (H-Care), UKM. Of these, 190 participants completed the 12-week intervention and were included in the final per-protocol analysis. Participants included both males and females. Although sex was recorded at baseline, the study was not powered to detect sex-specific effects of the intervention, and no stratified or interaction analyses by sex were conducted. Baseline demographic and clinical characteristics of participants across intervention groups are presented in Table 1. Participant recruitment, randomization, allocation, follow-up, and inclusion in the final analysis are summarized in Figure S1.

#### Inclusion criteria

a. Malaysian adults aged between 18-59 years old from the Klang Valley region
b. Moderate stress (scoring Perceived Stress Scale 10 within 14-26)
c. Scored Patient Health Questionnaire 9 (PHQ-9) less than or equal to 9
d. History of common cold symptoms within the past 6 months, assessed using the Jackson Cold Scale
e. Willing to comply with protocol requirements.

#### Exclusion criteria

a. Participant on immunosuppressant (i.e., steroids).
b. Use of immune-modifying medications or dietary supplements affecting the immune system (minimum 2-weeks washout required before inclusion).
c. Diagnosis of autoimmune disease or other significant health concerns.
d. Uncontrolled comorbidities including diabetes, hypertension, cardiovascular disease, chronic kidney disease, chronic liver disease, active malignancy, endocrine disorder and cancer.
e. Uncontrolled chronic respiratory illnesses including allergic rhinitis or asthma, and the presence of nasal ulcers/polyps.
f. Body temperature >38°C at study commencement.
g. Pregnant/lactating female.
h. Individuals following specific dietary requirements (diabetic diet/low salt diet/etc).
i. Current use of prebiotics or probiotics supplements (minimum 2 weeks washout required before inclusion).
j. Use of antibiotics within the past 2 weeks prior to enrolment.
k. Individuals with disabilities involving long-term physical, mental, intellectual and sensory impairment.
l. Individuals without symptoms of common colds within the past 6 months.
m. Known allergy to yeast, oat, wheat or barley.
n. Influenza vaccination within the past 1 year.
o. *Pneumococcal* vaccination within the past 5 years.
p. Participation in another research study within the same period.

#### Recruitment details

Potential participants were identified through: (1) advertisements posted at Universiti Kebangsaan Malaysia campus and Hospital Canselor Tuanku Muhriz; (2) announcements on social media platforms and university communication channels; (3) direct recruitment at health screening events conducted by the Centre for Healthy Ageing and Wellness. Interested individuals contacted the research team and underwent preliminary telephone screening to assess basic eligibility. Those meeting preliminary criteria were invited for an in-person screening visit where complete eligibility assessment, including physical examination, vital signs measurement, and blood tests, was performed. Approximately 300 individuals were screened to achieve the target enrollment of 198 participants. All participants provided written informed consent prior to study participation. The study protocol was approved by the relevant institutional ethics committee, and the trial was conducted in accordance with the Declaration of Helsinki and Good Clinical Practice guidelines.

### Method details

#### Randomization and blinding procedures

Eligible participants were randomly assigned in a 1:1:1 ratio to receive YBG 120 mg/day, YBG 204 mg/day, or an identical placebo. Randomization was computer-generated using GraphPad software by an independent biostatistician not involved in participant recruitment or assessment. The allocation sequence was concealed using sequentially numbered, opaque, sealed envelopes containing supplement codes (YBG-A, YBG-B, or YBG-C). These envelopes were held securely by the study enumerator at the Centre for Healthy Ageing & Wellness (H-CARE), who prepared and dispensed the supplements according to the randomization sequence. The enumerator had no contact with participants and was not involved in outcome assessments. The allocation key linking supplement codes to treatment groups was stored in a password-protected file accessible only to the independent biostatistician and was not revealed to investigators, participants, or outcome assessors until database lock and statistical analysis completion.

Blinding was thoroughly maintained across all trial stages. Study personnel involved in participant recruitment, data collection, and outcome assessments remained unaware of treatment allocation. Furthermore, laboratory staff analyzed blood samples blind to group assignment, using only participant IDs for sample identification. Research assistants administering questionnaires and clinical measures were also kept blinded. Data management, including entry and monitoring, was conducted by personnel without access to treatment codes. Although formal emergency unblinding procedures were established, no instances required their use. To prevent accidentally revealing the allocation concealment, a formal assessment of blinding success was not conducted.

#### Emergency unblinding

An emergency unblinding protocol was available for clinical situations in which knowledge of treatment assignment was necessary to guide medical care. No emergency unblinding occurred during the trial, and no withdrawals were due to supplement-related adverse events.

#### Intervention details

All supplement sachets containing either a placebo, YBG 120 mg or YBG 204 mg were manufactured to be indistinguishable from one another in appearance, handling, and sensory properties. Each 2g sachet consisted of an identically flavoured (mixed-berry) white granular powder with uniform texture and preparation instructions. To ensure consistent concealment, the supplements were prepackaged in uniform boxes labelled solely with a unique participant identification number and standardised consumption instructions. Participants consumed one sachet daily for 12 weeks, with the flexibility to take it in the morning or evening, either mixed with 20 ml of room temperature water or consumed directly. They were instructed to maintain their usual diet and lifestyle throughout the trial and to avoid starting any new supplements or medications without first notifying the research team.

#### Supplement composition

A complete nutritional and bioactive profiles are provided in Table 5.

**Table 5:** Nutrient and bioactive profile of yeast beta-glucan 1,3/1,6 and placebo sachets

	Group A (Placebo)	Group B (yeast beta-glucan supplement)	Group C (yeast beta-glucan supplement)
Active ingredient	Mixed berries juice powder Maltodextrin Sucralose Silicon dioxide	Wellmune yeast beta-glucan Mixed berries juice powder Maltodextrin Sucralose Silicon dioxide	Wellmune yeast beta-glucan Mixed berries juice powder Maltodextrin Sucralose Silicon dioxide
Ingredient declaration and percent range Dosage	NIL	100% Baker’s yeast beta-glucan 120 mg	100% Baker’s yeast beta-glucan 204 mg

Allergen information: The study product contained no detectable dairy, egg, fish, peanut, shellfish, soy, tree nut, or wheat derivatives.

#### Compliance monitoring and verification

Compliance was assessed using returned sachet counts, participant intake diaries, and regular follow-up by the research team. Empty and unused sachets were collected and counted at weeks 6 and 12, and daily diaries were used to document supplement consumption. Weekly follow-up via WhatsApp messaging and telephone calls was conducted to reinforce adherence and monitor adverse events. Compliance was calculated as the proportion of sachets consumed relative to those prescribed. Participants achieving ≥90% compliance (≥81 of 90 doses) were included in the per-protocol analysis, and reasons for non-compliance were documented.

#### Assessments procedures and timing

Evaluations were conducted at baseline, week 6, and week 12 for all study parameters. Safety-related blood parameters were assessed at baseline and at the final study visit (week 12).

#### Primary outcome

The primary outcome was upper respiratory tract infection (URTI) symptom severity, assessed using the validated Malay version of the Wisconsin Upper Respiratory Symptom Survey-21 (WURSS-21). The primary endpoint was defined as the change in the WURSS-21 total severity score from baseline to week 12 (day 90) reported in Table 2. The total severity score was derived from the sum of items 1-20, which measure both burden and symptom (items 1-10; e.g., runny nose, sore throat, cough, fatigue) and functional impact (items 11-20; e.g., ability to work, sleep, and perform daily activities). Item 21 (global severity rating) was recorded for descriptive purposes but was not included in the primary endpoint calculation. The total scores for the WURSS-21 range from 0 to 140, with higher scores meaning worse symptoms and less ability to function. The minimal clinically important difference (MCID) for the WURSS-21 is 6.5 points.

#### Secondary outcomes

Upper respiratory tract infection (URTI) occurrence, defined as the percentage of participants experiencing one or more URTI episodes during the 12-week period, was analyzed using the Chi-square test and is presented in Table 2. Fatigue was assessed using the Multidimensional Fatigue Inventory (MFI-20), with outcomes reported in Tables 2 and S3. Psychological mood status was measured via the Profile of Mood States questionnaire (POMS-40); changes in mood states are presented in Table 3, with corresponding outcomes in Figure 1 and Table S2. The Short Form-36 (SF-36) questionnaire was used to measure health-related quality of life, and the results are shown in Tables 3 and S4. All questionnaires (WURSS-21, POMS-40, MFI-20, SF-36) were provided in both Malay and English, allowing participants to select their preferred language or cross-reference between versions for clarity.

#### Additional assessment

Anthropometric assessments, including weight, height, BMI, and body composition (using a TANITA SC-330 bioimpedance analyzer), are reported in Table 1. Dietary intake was measured via a 3-day food record (encompassing 2 weekdays and 1 weekend day), which was subsequently analyzed using Nutritionist Pro software.^43^^,^^44^ Blood safety biomarkers, comprising a complete blood count, renal and liver function tests, fasting glucose, and a lipid profile, are presented in Table 4 and Figure 2, with additional detail in Table S5.

#### Safety monitoring

Safety was evaluated throughout the trial via assessments conducted at baseline, week 6, and week 12. These in-person visits included open-ended questioning regarding new symptoms or health concerns, measurement of vital signs (blood pressure, heart rate, temperature), and clinical chemistry and hematology blood testing at baseline and week 12. These procedures were supplemented by weekly telephone follow-ups to capture and monitor any interim adverse events or reported health changes.

#### Adverse event (AE) reporting procedures

All AEs were documented with onset date, duration, severity (mild, moderate, severe), relatedness to the intervention, and resolution status. Any serious adverse event (SAE) triggered immediate reporting (within 24 hours) to the principal investigator, institutional ethics committee, and study sponsor. Participants received emergency contact information and were instructed to report any unexpected symptoms promptly.

#### Prohibited concomitant medications

Participants were advised to avoid medications or supplements with potential immunomodulatory effects, including systemic corticosteroids, immunosuppressants, antibiotics (unless clinically indicated), probiotics, prebiotics, other beta-glucans, and high-dose vitamin C or D. Any use of prohibited agents was recorded and evaluated for possible influence on study outcomes.

### Quantification and statistical analysis

Data were analyzed using IBM SPSS 29 (Armonk, NY USA). Data normality was assessed using the Shapiro-Wilk test. Descriptive statistics are presented as mean ± standard deviation (SD) for continuous variables and frequency (percentage) for categorical variables. Between-group comparisons of baseline characteristics were conducted using one-way analysis of variance (ANOVA) for continuous variables and chi-square tests for categorical variables.^45^^,^^46^ For the primary and secondary outcome analyses, between-group differences and group × time interactions were evaluated using General Linear Model (GLM) repeated measures ANOVA. The GLM repeated-measures approach was selected for its ability to: (1) appropriately handle within-subject correlation across multiple timepoints (baseline, week 6, week 12); (2) test for group × time interactions, which directly address whether treatment effects differ over time; (3) adjust for baseline covariates including age and dietary intake variables (energy, carbohydrate, and protein) that showed baseline imbalances between groups; and (4) accommodate missing data patterns while maintaining statistical power. Mauchly’s test was used to assess the sphericity assumption, and Greenhouse-Geisser corrections were applied when sphericity was violated (ε < 0.75).

#### Sample size calculation

The required sample size for this trial was calculated using the randomized controlled trial formula proposed by Chan and Chan.^42^ The calculation was based on the primary endpoint of URTI symptom severity (measured by WURSS-21 total score), with reference parameters derived from effect sizes reported in a previous study that examined the effect of yeast beta-glucan on reducing URTI incidence in marathon runners (Mah et al., 2020),^14^ a population characterized by transient immune suppression. Although that study measured incidence, the effect sizes were used to estimate the expected reduction in symptom severity scores. This population was selected as the closest available proxy, as no prior human clinical trial had evaluated YBG for URTI symptom severity in moderately stressed adults. The sample size was determined using the following parameters: mean difference between intervention (0.38 ± 0.60) and control (0.52 ± 0.71) groups after 90 days of supplementation, standard deviation (σ) of the intervention group, and a constant (C) of 7.9 (corresponding to 80% power and 95% confidence interval). This design ensured adequate statistical power to detect clinically meaningful changes in URTI outcomes.

n = C / (μ^2^ – μ^1^) / σ)^2^ + 2)

n = 7.9/(0.24/0.60)^2^ + 2) ≈ 51 participants per arm.

In addition, allowing for a 30% dropout rate, the required sample size was increased to 66 participants per arm, resulting in a total of 198 participants across the three study arms. To achieve this, approximately 300 individuals were screened for eligibility.

#### Statistical significance and error control

The study was powered for the primary endpoint (WURSS-21 total severity score). Secondary outcomes including mood (POMS-40), fatigue (MFI-20), quality of life (SF-36), and biochemical markers were prespecified as exploratory. Consistent with this designation, no family-wise error rate adjustments were applied to avoid excessive Type II error risk. All p-values for secondary outcomes are therefore unadjusted and interpreted as hypothesis-generating. Statistical significance was set at α = 0.05 (two-tailed) for all analyses.

#### Covariate selection and adjustment rationale

Calorie, carbohydrate, and protein intake were included as covariates in the GLM based on their recognized role in modulating immune function and fatigue-related physiology.

(Calder et al., 2020),^37^ and because they exhibited small but statistically significant baseline differences between groups. This post hoc adjustment was implemented to reduce residual confounding, ensuring that observed changes in URTI symptoms, mood, and fatigue were more likely attributable to YBG supplementation than to background dietary variation.

It is important to note that these covariates were not pre-specified but were selected after unblinding due to the observed imbalances. While necessary to control for this confounding, post hoc covariate selection can inflate Type I error rates and introduce adjustment bias.^47^^,^^48^ This methodological choice is explicitly stated here to ensure analytical transparency.

#### Effect size estimation and confidence intervals

Effect sizes are reported as mean differences with associated 95% confidence intervals (CIs). CIs were computed using the pooled standard error from the GLM repeated-measures model, which accounts for within-subject covariance. For pairwise comparisons, CIs represent adjusted differences between each YBG dose and placebo, controlling for baseline scores and covariates (age, dietary energy, carbohydrate, and protein intake).

#### Missing data and analysis populations

##### Participant retention

Of 198 randomized participants, 190 completed the 12-week trial (96%). Eight participants discontinued (placebo: 3; YBG 120 mg: 3; YBG 204 mg: 2), all due to clinical reasons unrelated to the intervention (vaccination requirements, antibiotic use, or pregnancy). No withdrawals were related to adverse events or treatment-related issues (Figure S1).

#### Missing data handling

Participants who discontinued the study were excluded from the primary analyses. Given the low overall attrition rate (n = 8), no data imputation was performed. Sensitivity analyses to assess the potential impact of missing data were therefore not conducted. Consequently, all reported results are based on the observed data from study completers.

#### Analysis population

The primary analysis was conducted on a per-protocol (PP) population. This was defined as participants who completed all scheduled assessments at baseline, week 6, and week 12; achieved a compliance rate of at least 90% (consuming ≥81 of 90 doses); and had no major protocol deviations.

#### Rationale

A PP approach was chosen to evaluate biological efficacy requiring adequate exposure. High retention (96%), high compliance (>90%), and balanced baseline characteristics minimized bias. An ITT analysis was not conducted due to minimal missing data.

#### Compliance verification

Compliance was assessed using returned sachet counts, daily intake diaries, and weekly WhatsApp reminders.

#### Analysis populations and rationale for per-protocol approach

A per-protocol (PP) analysis was used as the primary analytical approach, including only participants who completed all three assessment timepoints, achieved ≥90% compliance, and had no major protocol deviations. This approach was appropriate because the study aimed to evaluate the biological efficacy of YBG under conditions of adequate exposure, and compliance was uniformly high across groups (>94%). Attrition was minimal (4%) and balanced, with no treatment-related discontinuations, reducing the risk of bias commonly associated with PP analyses. Baseline characteristics were comparable across groups, and dropouts occurred for logistical or personal reasons rather than intervention effects. An intention-to-treat (ITT) analysis for post-baseline outcomes was not feasible. While baseline characteristics were available for all randomized participants (Table S1), the eight participants who discontinued did not complete any post-baseline assessments, resulting in no outcome data for this group. Consequently, data imputation was deemed inappropriate given the trial’s exploratory nature and the resulting complete absence of follow-up data for dropouts. Taken together, the PP approach provides an internally valid assessment of dose-related efficacy while acknowledging that future effectiveness trials may require ITT analyses to capture real-world adherence patterns.^49^

### Additional resources

The complete study protocol detailing the overall trial design, eligibility criteria, interventions, and outcome measures was published in advance and is publicly accessible at https://doi.org/10.1136/bmjopen-2024-084277. Public access to the study registration details, including any protocol amendments, is provided through the clinical trial registry at ISRCTN 48336189.
