# Supplementary material for: Low-dose of yeast beta-glucan on respiratory symptoms and psychological well-being in moderately stressed adults
Source: iScience. 2026 Feb 25;29(3):115120. doi: 10.1016/j.isci.2026.115120 (PMC13010125; doi:10.1016/j.isci.2026.115120)
Supplement: Document S1. Figures S1, Tables S1–S5, and Data S1 [file mmc1.pdf]

iScience, Volume *29*

Supplemental information

Low-dose of yeast beta-glucan on respiratory symptoms and psychological well-being in moderately stressed adults

Nur Nadia Mohamad Habibullah, Munirah Ismail, Norhayati Ibrahim, Shirley Gee Hoon Tang, Hanif Farhan Mohd Rasdi, Mohamed Faisal Abdul Hamid, Kalavathy Ramasamy, So Fie Tan, and Suzana Shahar

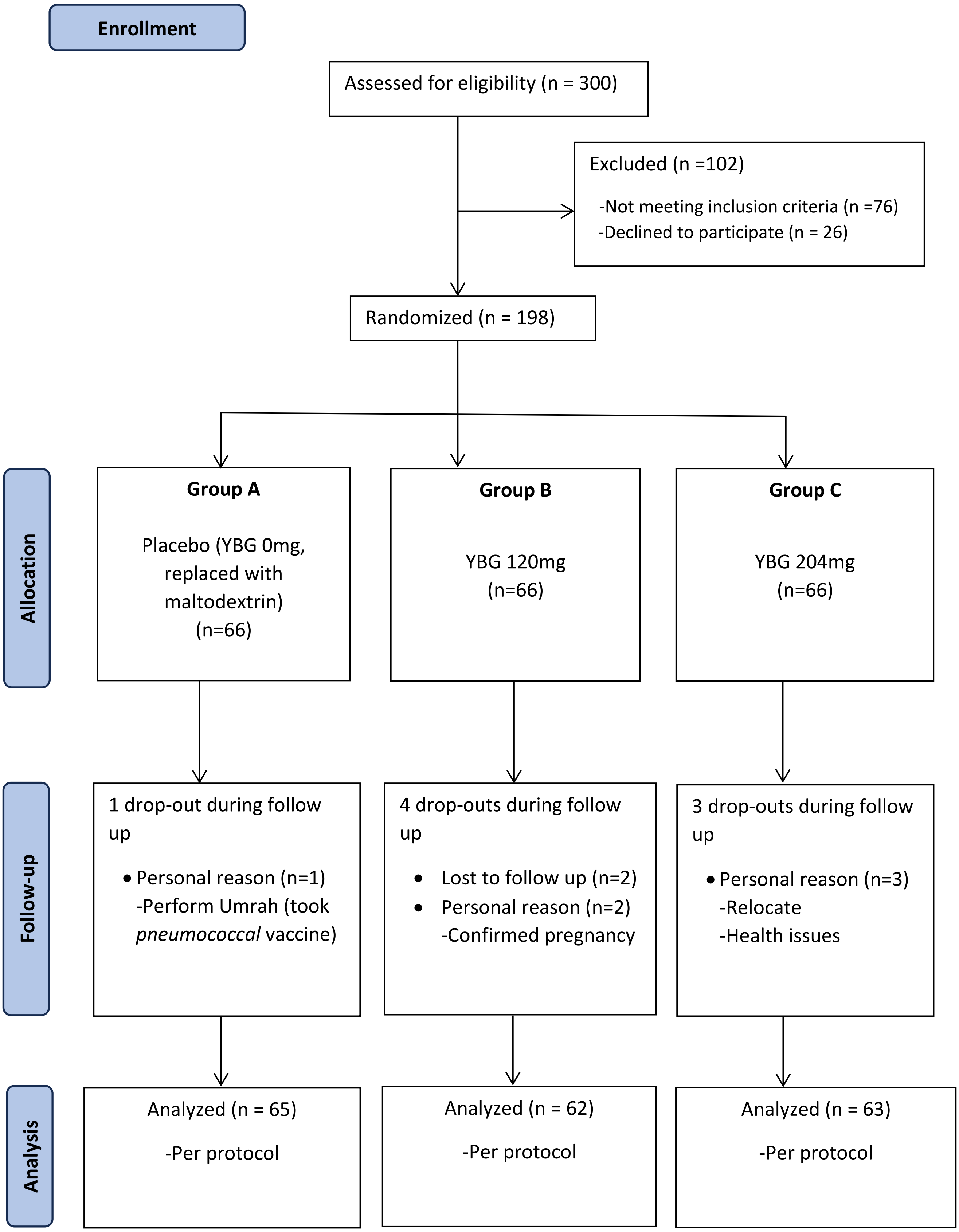

Figure S1 Study recruitment and participant flow diagram

Table S1 Comparison of baseline characteristics between completers (n=190) and dropouts (n=8)

Baseline comparison of sociodemographic and anthropometric between intervention and placebo groups [completers (n=190)]

Characteristics	Placebo (n=65)	YBG 120mg (n=62)	YBG 204mg (n=63)	Total (n=190)	p-Value
**Sociodemographic Variables**					
**Age, mean ± SD**	27.85 ± 9.0	25.52 ± 6.7	29.35± 9.87	27.57±8.7	0.038
**Gender**					
Male	23 (34.8)	19 (28.8)	25 (37.9)	67 (33.8)	. 0.536
Female	43 (65.2)	47 (71.2)	41 (62.1)	131 (66.2)	
**Race n (%)**					
Malay	47 (71.2)	52 (78.8)	41 (62.1)	140 (70.7)	0.438
Non-malays	19 (28.8)	14 (21.2)	25 (37.9)	58 (29.3)	
**Marital Status n (%)**					
Single	48 (72.7)	55 (83.3)	45 (68.2)	148 (74.7)	0.164
Married/Widow/Divorce	18 (27.3)	11 (16.7)	21 (31.8)	50 (25.3)	
**Educational Level n (%)**					0.790
Certificate/Diploma/Bachelor	57 (32.4)	59 (33.5)	60 (34.1)	176 (88.9)	
Masters/PhD	9 (40.9)	7 (31.8)	6 (27.3)	22 (11.1)	
**Employment Status n (%)**					
Student	44 (32.6)	49 (36.3)	42 (31.1)	135 (68.2)	0.526
Employed	22 (34.9)	17 (12.6)	24 (38.1)	63 (31.8)	
**Alcohol Status n (%)**					
Yes	7 (10.6)	10 (15.2)	6 (9.1)	23 (11.6)	0.532
No	59 (89.4)	56 (84.8)	60 (90.9)	175 (88.4)	
Weight (kg) mean ±SD	62.32 ± 15.35	64.24 ± 14.32	63.55 ± 13.62	63.37±14.40	0.742
BMI (kg/m2) mean ±SD	24.06 ± 5.49	24.37 ± 5.34	24.23 ± 4.64	24.22 ± 5.15	0.943
Percentage Body Fat (%) mean ±SD	32.38 ± 8.83	32.92 ± 10.32	31.62 ± 9.92	32.31 ± 9.68	0.744
Skeletal muscle mass (kg) mean ±SD	22.45 ± 5.09	23.39 ± 5.60	23.85 ± 5.69	23.23 ± 5.47	0.326
Blood Pressure Systolic mean ±SD (mmHg)	109.08 ± 13.59	109.26 ± 13.07	111.55 ± 12.19	109.96 ± 12.95	0.474
Blood Pressure Systolic (mmHg) mean ±SD	71.17 ± 8.23	72.33 ± 9.16	73.62 ± 8.39	72.37 ± 8.62	0.263

Data were presented as *n* (%) or mean ± standard deviation (SD). * Significant value at p<0.05 using the One-Way ANOVA for continuous variables

Baseline comparison of sociodemographic and anthropometric between intervention and placebo groups [dropouts (n=8)]

**Characteristics**	**Placebo (n=66)**	**YBG 120mg (n=66)**	**YBG 204mg (n=66)**	**Total (n=198)**	**p-Value**
**Sociodemographic Variables**					
Age, mean ± SD	27.9 ± 9.0	25.4 ± 6.9	29.6 ± 10.0	27.03 ± 8.67	*0.029
**Gender**					
Male	22 (33.3)	22 (33.3)	22 (34.8)	67 (33.8)	0.756
Female	44 (66.7)	44 (66.7)	43 (65.2)	131 (66.2)	
**Race n (%)**					
Malay	29 (44.6)	28 (43.8)	35 (50.7)	92 (46.7)	0.269
Non-malays	36 (55.4)	36 (56.3)	34 (49.3)	106 (53.5)	
**Marital Status n (%)**					
Single	48 (73.8)	53 (79.1)	49 (74.2)	150 (75.8)	0.176
Married/Widow/Divorce	17 (26.2)	14 (20.9)	17 (25.8)	48 (24.2)	
**Educational Level n (%)**					
Certificate/Diploma/Bachelor	56 (86.2)	56 (86.2)	57 (87.7)	169 (85.4)	0.744
Masters/PhD	9 (13.8)	9 (13.8)	11 (12.3)	29 (14.6)	
**Employment Status n (%)**					
Student	43 (66.2)	46 (65.7)	40 (63.5)	129 (65.2)	0.411
Employed	22 (33.8)	24 (34.3)	23 (36.5)	69 (34.8)	
**Alcohol Status n (%)**					
Yes	9 (13.2)	10 (16.1)	10 (14.7)	29 (14.6)	0.485
No	59 (86.8)	52 (83.9)	58 (85.3)	169 (85.4)	
**Anthropometry, mean ± SD**					
Weight (kg), mean ± SD	62.1 ± 15.4	64.0 ± 14.7	63.6 ± 13.8		0.749
BMI (kg/m^2), mean ± SD	24.0 ± 5.5	24.3 ± 5.5	24.3 ±4.7		0.947
Percentage Body Fat **(%)**, mean ± SD	32.5 ± 8.9	32.8 ± 10.6	32.0 ± 9.9		0.905
Skeletal Muscle Mass (kg), mean ± SD	22.3 ± 5.0	23.3 ± 5.7	23.3 ± 5.3		0.477
Blood Pressure					
Blood Pressure Systolic (mmHg) mean ± SD	109.1 ± 13.7	108.6 ± 13.2	111.5 ± 12.5		0.423
Blood Pressure Diastolic (mmHg) mean ± SD	71.1 ± 8.3	71.9 ± 9.2	73.5 ± 8.6		0.272

Data were presented as *n* (%) or mean ± standard deviation (SD). * Significant value at p<0.05 using the One-Way ANOVA for continuous variables

Table S2 Mean changes in profile of mood states (poms) scores from baseline to weeks 6 and 12

Parameter	Mean Change ± SD			
	Placebo (n=65)	YBG 120mg (n=63)	YBG 204mg (n=62)	p-value
**TENSION**				
Baseline - Week 6	-0.40 ± 4.06	-0.48 ± 5.14	-0.08 ± 4.89	.77
Baseline - Week 12	4.06 ± 5.91	-1.03 ± 4.61	-0.19 ± 4.37	<.00**
**DEPRESSION**				
Baseline - Week 6	-0.46 ± 4.30	-0.61 ± 4.72	-0.56 ± 4.94	.98
Baseline - Week 12	2.37 ± 5.35	-0.71 ± 4.18	-0.56 ± 5.06	<.00**
**ANGER**				
Baseline - Week 6	-0.37 ± 3.27	-1.55 ± 4.00	-1.08 ± 3.80	.20
Baseline - Week 12	1.00 ± 3.50	-1.47 ±3.55	-1.08 ± 3.40	<.00**
**FATIGUE**				
Baseline - Week 6	-0.15 ± 3.94	-1.13 ± 5.33	-0.73 ± 5.02	.52
Baseline - Week 12	1.46 ± 4.26	-1.31 ± 4.48	-1.25 ± 5.05	<.00**
**CONFUSION**				
Baseline - Week 6	-0.46 ± 3.89	-1.27 ± 4.66	-1.03 ± 4.01	.53
Baseline - Week 12	-0.34 ± 3.76	-2.00 ± 4.58	-1.30 ± 4.09	.08
**VIGOUR**				
Baseline - Week 6	0.28 ± 5.50	0.73 ± 6.32	-0.44 ± 6.72	.57
Baseline - Week 12	0.03 ± 7.11	0.50 ± 7.28	-0.37 ± 5.92	.78
**TMD NEGATIVE**				
Baseline - Week 6	-1.85 ± 16.76	-5.05 ± 20.49	-3.32 ±19.81	.64
Baseline - Week 12	8.55 ± 19.04	-6.52 ±17.34	-4.38 ±19.81	<.00**
**TMD POSITIVE**				
Baseline - Week 6	1.12 ± 7.56	1.13 ± 8.68	-0.29 ± 8.73	.50
Baseline - Week 12	1.06 ± 9.40	1.34 ± 9.57	0.21 ± 7.65	.76
**TOTAL POMS**				
Baseline - Week 6	-2.12 ± 16.33	-5.77 ± 23.27	-2.87 ± 21.14	.57
Baseline - Week 12	8.52 ± 19.93	-7.02 ± 20.45	-4.02 ± 21.80	<.00**

Table S3 Mean changes in multidimensional fatigue inventory from baseline to weeks 6 and 12

Parameter	Mean Change ± SD			
	Placebo (n=65)	YBG 120mg (n=63)	YBG 204mg (n=62)	p-value
**Reduced activity**				
Baseline - Week 6	-0.52 ± 3.63	-1.02 ± 4.23	0.25 ± 4.36	.22
Baseline - Week 12	-0.40 ± 3.83	-0.56 ± 4.87	-0.46 ± 4.62	.98
**Reduced motivation**				
Baseline - Week 6	-0.32 ± 2.91	-0.73 ± 4.01	-0.02 ± 4.33	.58
Baseline - Week 12	-0.42 ± 3.83	-1.06 ± 4.35	-0.71 ± 3.80	.66
**Mental fatigue**				
Baseline - Week 6	-0.38 ± 3.77	-0.87 ± 4.57	-0.49 ± 4.46	.80
Baseline - Week 12	-0.59 ± 4.03	-0.69 ± 5.01	-0.98 ± 3.86	.87
**General fatigue**				
Baseline - Week 6	-0.06 ± 3.62	-0.60 ± 4.77	-0.25 ± 4.82	.79
Baseline - Week 12	-0.32 ± 3.67	-0.71 ± 4.18	-0.43 ± 3.93	.85
**Physical fatigue**				
Baseline - Week 6	-0.75 ± 3.61	-0.27 ± 4.63	-0.67 ± 5.43	.82
Baseline - Week 12	-0.95 ± 3.97	-0.40 ± 3.91	-0.61 ± 4.31	.73

Table S4 Mean changes in quality of life (SF-36) from baseline to weeks 6 and 12

Parameter	Mean Change ± SD			
	Placebo (n=65)	YBG 120mg (n=63)	YBG 204mg (n=62)	p-value
**PF**				
Baseline - Week 6	-1.69 ± 25.18	1.21 ± 25.49	6.91 ± 30.05	.19
Baseline - Week 12	-1.00 ± 24.15	4.19 ± 23.49	9.21 ± 25.34	.06
**RLP**				
Baseline - Week 6	-0.77 ± 30.29	3.23 ± 39.59	11.51 ± 38.05	.15
Baseline - Week 12	-0.39 ± 34.66	6.05 ± 38.59	12.70 ± 38.86	.14
**Pain**				
Baseline - Week 6	-0.69 ± 25.91	3.07 ± 26.42	8.37 ± 27.38	.16
Baseline - Week 12	3.27 ± 22.97	4.64 ± 25.07	13.06 ± 23.68	.05
**GH**				
Baseline - Week 6	2.15 ± 19.88	3.07 ± 27.09	-1.11 ± 19.52	.55
Baseline - Week 12	5.39 ± 20.35	5.08 ± 26.08	4.05 ± 19.61	.94
**RLE**				
Baseline - Week 6	6.67 ± 51.10	12.37 ± 42.78	13.23 ± 51.69	.71
Baseline - Week 12	13.33 ± 43.22	3.76 ± 50.90	15.34 ± 48.94	.35
**EF**				
Baseline - Week 6	5.38 ± 23.24	2.90 ± 25.68	-0.64 ± 23.08	.37
Baseline - Week 12	5.39 ± 21.98	4.03 ± 21.590	-0.16 ± 24.49	.36
**EW**				
Baseline - Week 6	4.25 ± 20.19	5.94 ± 21.62	-0.51 ± 21.63	.21
Baseline - Week 12	3.94 ± 18.68	6.58 ± 22.58	0.83 ± 21.67	.31
**SF**				
Baseline - Week 6	1.35 ± 27.07	4.44 ± 36.01	9.52 ± 25.57	.30
Baseline - Week 12	6.73 ± 28.48	5.04 ± 35.10	11.51 ± 27.71	.47
**PHC**				
Baseline - Week 6	1.22 ± 68.06	10.56 ± 84.36	24.64 ± 78.86	.23
Baseline - Week 12	10.11 ± 75.66	19.96 ± 73.87	39.33 ± 75.00	.08
**MHC**				
Baseline - Week 6	18.80 ± 83.04	25.64 ± 96.01	21.07 ± 95.39	.91
Baseline - Week 12	29.39 ± 87.23	19.42 ± 103.91	27.58 ± 99.17	.83

Table S5 Mean changes for blood safety parameters from baseline to weeks 6 and 12

Parameter	Mean Change ± SD			
	**Placebo (n=65)**	**YBG 120mg (n=63)**	**YBG 204mg (n=62)**	**p-value**
**Total Protein**				
Baseline - Week 12	-1.00 ± 4.10	-0.97 ± 4.05	-1.29 ± 3.84	.89
**Albumin**				
Baseline - Week 12	-0.65 ± 2.48	-0.62 ± 2.41	-0.38 ± 2.93	.83
**Globulin**				
Baseline - Week 12	-0.35 ± 3.40	-0.44 ± 3.18	0.94 ± 3.21	.56
**Urea**				
Baseline - Week 12	0.04 ± 1.02	-0.14 ± 1.09	-0.02 ± 9.32	.65
**Creatinine**				
Baseline - Week 12	-1.12 ± 6.79	-1.05 ± 7.99	-2.03 ± 6.20	.68
**eGFR**				
Baseline - Week 12	1.54 ± 9.08	0.74 ± 9.97	1.92 ± 6.44	.74
**Uric Acid**				
Baseline - Week 12	0.31 ± 51.82	-25.69 ± 50.44	-12.24 ± 54.38	.02*
**Sodium**				
Baseline - Week 12	-0.75 ± 2.24	-0.03 ± 2.47	-0.46 ±3.64	.36
**Potassium**				
Baseline - Week 12	0.23 ± 0.57	0.06 ± 0.64	0.27 ± 0.64	.14
**Chloride**				
Baseline - Week 12	-0.65 ± 1.91	-0.79 ± 1.72	-0.86 ± 1.61	.78
**HRCP**				
Baseline - Week 12	-0.37 ± 2.44	-0.29 ± 1.24	-1.14 ± 3.54	.13
**TCH**				
Baseline - Week 12	-0.10 ± 0.56	-0.17 ± 0.55	-0.08 ± 0.50	.60
**HDL**				
Baseline - Week 12	0.01 ± 0.21	-0.01 ± 0.14	-0.02 ± 0.19	.64
**LDL**				
Baseline - Week 12	-0.11 ± 0.42	-0.11 ± 0.42	-0.08 ± 0.46	.92
**TG**				
Baseline - Week 12	-0.01 ± 0.26	-0.06 ± 0.26	0.02 ± 0.37	.31
**Total HDL/Cholesterol Ratio**				
Baseline - Week 12	-0.05 ± 0.38	-0.08 ± 0.29	0.03 ± 0.47	.27
**Glucose**				
Baseline - Week 12	-0.17 ± 0.38	-0.15 ± 0.64	-0.09 ± 0.48	.63
**HBA1c**				
Baseline - Week 12	-0.17 ± 0.56	-0.19 ± 0.32	-0.16 ± 0.30	.90

Data S1: Psychometric validation dataset and statistical analysis for WURSS-21_M

Translation and Cultural Adaptation

The Wisconsin Upper Respiratory Symptom Survey-21 (WURSS-21) was translated into Malay following a standardized forward-backward translation process in accordance with the COSMIN study design checklist. An expert panel comprising respiratory physicians and clinical psychologists evaluated the translated version to ensure conceptual accuracy and semantic equivalence with the original English instrument. Cognitive debriefing was conducted with 30 participants from the target population to assess item clarity, cultural relevance, and comprehensibility. Pre-testing confirmed that all items were clearly understood by Malay-speaking adults, supporting the cultural appropriateness of the Malay-adapted instrument (WURSS-21_M).

Participants and Data Collection

Psychometric validation was conducted in a sample of 150 Malay-speaking adults presenting with upper respiratory tract symptoms. Participants were aged 18-59 years and were recruited from the Klang Valley, Malaysia, corresponding to the same geographic region as the intervention trial. The questionnaire was administered in a culturally adapted Malay format, with bilingual support provided when required.

Reliability

Internal consistency reliability of the WURSS-21_M was excellent, with a Cronbach's alpha coefficient of 0.92. This value exceeds the recommended threshold of 0.70 for health outcome measures and indicates strong inter-item correlations within the scale.

Construct Validity

Construct validity was evaluated through concurrent validity analysis with the Profile of Mood States (POMS-40). WURSS-21_M total scores demonstrated a significant negative correlation with POMS-40 scores ($r = -0.261$, $p = 0.001$), consistent with theoretical expectations that greater upper respiratory symptom burden is associated with poorer mood states. This finding supports the construct validity of the Malay version of the instrument.

Factor Structure

Exploratory factor analysis supported the structural validity of the WURSS-21_M. Sampling adequacy was confirmed by a Kaiser-Meyer-Olkin (KMO) measure of 0.862, and Bartlett's test of sphericity was significant ($p < 0.001$), indicating suitability for factor analysis. A four-factor solution was identified, comprising functional limitation, throat and chest symptoms, nasal symptoms, and illness perception. All items demonstrated satisfactory factor loadings (>0.40) on their respective primary factors.

Summary

Together, these findings support the view that the WURSS-21_M exhibits strong internal consistency and satisfactory construct validity, with a factor structure broadly consistent with the original instrument. The Malay-adapted WURSS-21 therefore indicates potential as a reliable and culturally appropriate tool for assessing upper respiratory symptom severity and functional impact among Malay-speaking adults in Malaysia.
